# Views of general practitioners on end-of-life care learning preferences: a systematic review

**DOI:** 10.1186/s12904-022-01053-9

**Published:** 2022-09-21

**Authors:** Shrikant Atreya, Soumitra S. Datta, Naveen Salins

**Affiliations:** 1 Department of Palliative Care and Psychooncology, Tata Medical Center, Kolkata, West Bengal 700160 India; 2 Institute of Clinical Trials and Methodology, University College London, London, UK; 3 Department of Palliative Medicine and Supportive Care, Kasturba Medical College, Manipal Academy of Higher Education, Manipal, India

**Keywords:** General practitioner, Continuing medical education, End of life care, Motivation

## Abstract

**Background:**

General practitioners (GPs) play a pivotal role in providing end-of-life care in the community. Although they value end-of-life care, they have apprehensions about providing care in view of the limitations in knowledge and skills in end-of-life care. This review aimed to explore, synthesise, and analyse the views of general practitioners on end-of-life care learning preferences.

**Methods:**

MEDLINE, CINAHL, PsycINFO, EMBASE, Scopus, Web of Science, and Cochrane were searched for literature on the views of general practitioners on end-of-life care learning preferences from 01/01/1990 to 31/05/2021. Methodological quality was reported.

**Results:**

Of the 10,037 articles identified, 23 were included for the review. Five themes developed from the review. The desire to provide palliative care, as well as self-actualisation needs, relevance to practice, a sense of responsibility, and a therapeutic bond, motivates general practitioners to learn end-of-life care. Some of the learning needs expressed were pain and symptom management, communication skills, and addressing caregiver needs. Experiential learning and pragmatist learning styles were preferred learning styles. They perceived the need for an amicable learning environment in which they could freely express their deficiencies. The review also identified barriers to learning, challenges at personal and professional level, feelings of disempowerment, and conflicts in care.

**Conclusion:**

GPs’ preference for learning about end-of-life care was influenced by the value attributed to learning, context and content, as well as preference for learning styles and the availability of resources. Thus, future trainings must be in alignment with the GPs’ learning preferences.

**Supplementary Information:**

The online version contains supplementary material available at 10.1186/s12904-022-01053-9.

## Key messages

1. **What is already known?**
  A) General practitioners play a pivotal role in delivering primary palliative care to patients in the community. This helps in a decentralised approach to palliative care delivery which could serve large population of patients in need of it.
  B) There is substantial evidence that general practitioners provide palliative care. Studies have explored the following: various primary palliative care models, barriers and facilitators in primary palliative care provision, educational interventions through continuing professional developments of GPs in end-of-life care and the challenges of providing palliative care by rural GPs
2. **What are the new findings?**

GP’s learning preferences in end-of-life care are influenced by the value that they attribute to learning and how empowered they feel in providing end-of-life care.

Experience in clinical practice significantly influences their learning preference.

Many GPs prefer to learn by reflecting on their clinical practice under the guidance of specialists or experienced GP colleagues.

They prefer the learning environment to be sensitive to their needs, non-intimidating and trustworthy where they could democratically exchange their challenges in knowledge and skills in end-of-life care

3. **What is the significance?**

Future training programs in end-of-life care could be implemented in alignment with the learning preferences of GPs. The training programs must factor the facilitators and barriers in attending training programs and training styles that best fit their learning needs and fulfills their learning requirements.

## Introduction

General practitioners (GPs) assume a central role in delivering end-of-life care in the community [1]. They provide care in alignment with the patient’s wishes, coordinate care with the multidisciplinary team, and prevent unnecessary hospital admissions [2–4]. Studies have revealed that despite having played a key role in care provision, GPs felt ill-equipped and lacked confidence in end-of-life care provision [5]. They particularly perceived managing physical symptoms, addressing patients’ and caregivers’ psychological needs, and advance care planning as a daunting experience [1]. GPs prefer to undergo training in End-of-Life care in order to keep themselves abreast of the growing body of evidence and changing guidelines [1]. These factors may influence end-of-life care provision and mandates an urgent evidence-based training programs in end-of-life care in alignment with GPs needs.

GPs access training only if the training program honours their past experience, has relevancy to their clinical practice and adds valuable information to address the gap in their knowledge [6, 7]. The GP’s preference for End-of-life care learning was analysed using the social constructivist learning theory [8–12]. Social constructivist learning theory is built on the premise that learning is a cognitive process that is constructed through social interaction [8–12]. The theory emancipates the learner by emphasising on the importance of learning as being self-directed with learners having the locus of control [8–12].

A scoping review using a multidisciplinary database, SCOPUS [13] identified previous systematic reviews on end-of-life care provision by GPs. They explored barriers and facilitators in palliative care provision [14–16], models that supported GPs’ provision of end-of-life care [17], continuing professional developments for GPs in end-of-life care [18], the role and performance of GPs in end-of-life care [19], and the challenges of providing palliative care by rural GPs [20]. Although there are studies that have explored GP’s views on end-of-life care learning preferences, these have not been systematically synthesised and analysed, necessitating the conduct of this review.

## Literature review methods

### Aim of the review

The aim of this systematic review was to explore, synthesise, and analyse the views of GPs on end-of-life care learning preferences.

### Review question

What are the views of GPs on end-of-life care educational needs and learning preferences?

The end-of-life care learning preferences of GPs were reviewed using the Population, Phenomenon of Interest, and Context (PICo) framework [21]. The population studied was GP, the phenomenon of interest was views on learning preferences and the context was end-of-life care.

### The philosophical paradigm and theoretical framework of research

Constructivist grounded theory was the philosophical paradigm underpinning this review that explored the GP’s experiential views, their actions and interactions, and the implicit meanings attributed to their learning preferences [22]. Social constructivist learning theory was applied to understand how individuals construct knowledge in a social context [8–12]. It is built on three premises: cognitive processing of knowledge, self-directed learning, and social construction of knowledge.

### Review design

A scoping search was conducted to determine the breadth of the evidence available on the phenomenon explored. The search showed a heterogeneous mixture of quantitative and qualitative studies. Popay’s narrative synthesis method enables synthesis of the data from a mixed typology of studies into themes [23]. Moreover, it allows using a theoretical framework for the interpretation of review findings and provides flexibility in choosing the methods within each step of the synthesis relevant to the review.

### Search strategy

The review question was divided into search concepts, which were further used to conduct an initial scoping review. Scoping review helped derive key search terms relevant to each concept of the review questions. The search identified three papers to test the sensitivity of the search [24–26]. The search terms identified from these studies were further expanded into thesaurus terms and free text terms [27]. A search was conducted using electronic databases (MEDLINE, EMBASE, CINAHL, and PsychINFO) to identify articles published in English between 01/01/1990 and 31/05/2021 (Additional file 1), as the first article on palliative care was published in MEDLINE in 1993 [28]. The search was conducted using thesaurus and free-text terms specific to the database, and the terms were combined using Boolean operators [27]. Additionally, searches were conducted using SCOPUS, the Web of Science, and the Cochrane database using free texts. A list of 12 journals was hand searched for additional citations (Additional file 2). The bibliographies of the full-text articles were screened using the Google Scholar database for any new articles that could be added.

### Study eligibility

Selection criteria of studies included is provided Table 1.

**Table 1:** Selection Criteria of the Studies included in the review

**Inclusion Criteria:** 1. Studies published in English from 01/01/1990 onwards 2. Studies that explored views of general practitioner or similar healthcare provider on end of life care learning preferences 3. Community/Home care setting 4. Studies with Hawker’s methodological quality score of 19 or more.	**Exclusion criteria**: 1. Learning preferences other than end of life care 2. Medical practitioners other than general practitioners or family physicians 3. Studies conducted in a hospital or hospice setting

### Assessing methodological rigor of the studies included in the review

As the review included a mixture of qualitative and survey studies, Hawker’s tool was used to assess its methodological rigour [29]. There are a growing number of palliative care systematic reviews that have used this tool [30–32]. Hawker’s tool allows systematic appraisal of the study by analysing the title and abstract, introduction and objectives, method and data, sampling, data analysis, ethical aspects, results, transferability/generalizability, and implications of the study [29]. These criteria were scored between 1 and 4 (1 = very poor and 4 = good). A score of 9 was considered a minimum score and a score of 36 as a maximum (Additional file 3) [29]. Although Hawker does not mention a cut-off score, based on the previous studies [31, 33], a cut-off was set at 19. Three studies were excluded from the review as they had a score of less than 19 [34–36]. The minimum score of the studies included in the review was 19 and the maximum score was 32. Furthermore, the studies were classified into the following grades: “high quality” (A), 30–36 points; “medium quality” (B), 24–29 points; and + "low quality” (C), 19–23 points [37]. Six studies were of high quality [24–26, 38–40], ten studies were of medium quality [41–50] and seven studies were of poor quality [51–57].

### Data extraction

Screening, quality appraisal, and data extraction were conducted independently by two reviewers. The third reviewer helped resolve the conflicts. The initial section of the data extraction sheet had information regarding the country and year of publication. The second section focused on the type of study, that is, survey, qualitative, or mixed-method. In this section, study objectives, population, and study setting were also described. The study sample, participants, inclusion and exclusion criteria, research design, and methods were elucidated in the third section. The fourth section provided information on the study findings and conclusions.

### Data synthesis

The review findings were synthesised using Popay’s narrative synthesis [23]. The first step of the narrative synthesis is to identify a theoretical framework, and social constructivist learning theory was used to interpret the findings of the review [9]. It was followed by developing a preliminary synthesis that involved a brief description of the studies in the review. The data gathered was classified into countries, the year of publication, type of population, and the factors involved in constructing knowledge. The words and texts extracted helped the reviewers familiarise with the study findings before analysis. Patterns were identified from the preliminary synthesis, and the reviewers explored the relationships within and between studies in order to generate meaningful categories and themes. The reviewers were mindful of gleaning the similarities and differences in the data generated. The fourth step was to assess the robustness of the synthesis. To ensure this, the reviewers critically reflected on the synthesis process and identified possible sources of bias [23].

### Data analysis using social constructivist learning theory

Social constructivist learning theory explicates that knowledge is constructed through social interaction. Thus, using this theory, researcher will interpret the following using social constructivism theory: value that general practitioners attribute to End-of-life care learning and factors that facilitate and impede learning. Social constructivism asserts that learning is an inherently social process rather than a mere acquisition and assimilation of facts and figures, thus helping interpret various styles that GPs adopt in learning end-of-life care.

### Review findings

#### Overview of the studies

**Fig. 1:**
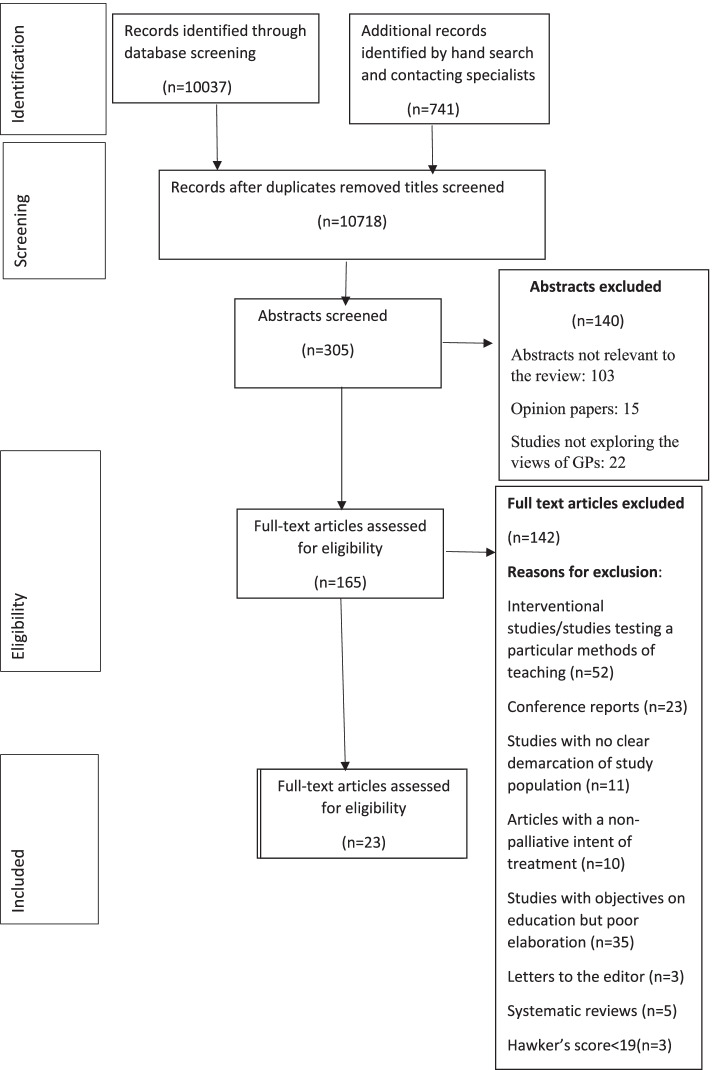
PRISMA Flow Diagram

Out of 10,037 articles identified from the database searches, 23 articles were included for synthesis (The PRISMA flow diagram**-**Fig. 1). Eleven studies were qualitative, eleven were surveys, and one was a mixed-method study. Fifteen studies were from Europe (eight from the United Kingdom, two from Belgium, and one each from Denmark, the Netherlands, Austria, Germany, and Ireland), six from Australia, and two from Canada. The qualitative studies were single centric and quantitative studies were combination of single centric and multicentric studies (Table 2). Two studies included combination of GPs and paediatricians involved in general practice [43, 49]. In United Kingdom, Europe, Australia and Canada, the health care system heavily relies on gatekeeping by GPs. The health care system provides universal coverage of health insurance and mandates gatekeeping by GPs in order for patients to access insurance facility. In some countries such as Canada, specialists receive less payment for non-GP referred patients. This could perhaps justify the reason for these countries to have studies on end-of-life care provision by GPs ([58], https://www.commonwealthfund.org/sites/default/files/2020-12/International_Profiles_of_Health_Care_Systems_Dec2020.pdf).

**Table 2:** Overview of the Studies included in the Systematic Review

Author (Year) Country	Research question	Participant/setting	Method	Key findings	Hawker score
Selman et al. (2017) [24] London, UK	The study aimed to explore GP’s educational needs, preferred learning methods, and acceptable methods of evaluation.	28 General practitioners (18 trainees; 10 General practitioners)	Qualitative data analysis	**Motivation to learn**: Generalists must be specialist at end-of-life care, Attrition of skills and with symptom management and use of syringe drivers due to inconsistent exposure, (Ability to) deal with complexity of end-of-life care. **Learning needs: S**ymptom management, need for identifying local palliative care resources, and communication skills caring for patients with non-malignant conditions; and paediatric palliative care. To handle difficult conversation around denial and bargaining, handle emotionally burdensome, care in alignment with patient’s wishes. **Learning style**: They preferred experiential learning in a mentored environment from palliative care specialists/experienced general practitioners, most preferred placements at hospices and opportunities to shadow or discussion of cases with specialist palliative care team, Also learning from relatives/family members of patients. Case studies preferred over didactic learning style; E-learning had mixed views while it was cost effective and flexible **Facilitators of training**: They preferred multi-disciplinary mentors and peers such as specialist palliative care/GP mentors **Feedback preference**: Behavioural assessment using videotaping or even simulated behaviour assessment was not effective Self-assessment questionnaire was of limited use if it was not lengthy or burdensome. They were in favour of patient and family feedback if done sensitively, some felt inappropriate to ask a feedback from a relative about their general practitioner who looked after their dying patient Timing patient and family outcome measurement sensitively was considered important. **Barriers for learning**: When specialists lacked trust in general practitioners.	**32**
Pype at al. (a) (2014) [25] Belgium	The study explored current experiences of General practitioners, CME providers and PHCT members with palliative-care education for General practitioners Their views on and preferences for future palliative-care education for General practitioners according to General practitioners, CME providers and PHCT members respectively	29General practitioners	Qualitative research method using Focus group discussion	**Motivation for learning:** General practitioners felt palliative care as a part of their job; Felt the need for a shift in attitude from “cure to care” to look beyond the usual framework of a diagnosis, a therapy, making somebody better. Self-conception, roles and responsibilities were perceived by the practitioner Desired life-long learning for knowledge updation and to prevent volatility. **Learning needs**: Preferred to remain generalists, keeping patients free of pain and discomfort, up scaling their communication skills, **Learning style**: General practitioners were not enthusiastic about CMEs as they felt it was theoretical and did not match their actual training needs. Better way to address their needs were by workplace learning, trial and error, learning by doing, reflecting on one’s practice and being mentored by specialists. Learning by the bedside under the supervision of specialists They also felt learning from carers as equally important. Co-manage a patient with palliative home care teams helped learn to shift from reactive style to a more proactive style. Case-based interactive discussions and peer discussions, had better and prolonged retention of information. They expressed the need for experiential learning by practising and training in small groups, and role-play, and with simulated patients and with inputs from specialists as an ongoing process. They felt benefited from planning meetings as it involved professionals and exchange of ideas. They preferred case-based discussion in small groups. **Facilitators of learning**: Sharing of experience with peers in the group, mentorship by specialists Feedback on action and doing from specialists and peers. They needed a respectful (safety and trust), non-intimidating and non-judgemental learning environment, off bedside not exposing their deficiencies to patient/family **Barriers of learning:** Limited involvement in palliative care, care as time consuming and emotionally exhausting, lack of clarity in roles and responsibilities, felt inhibited when the care of their patients was taken over by specialists. Challenges of not being able replicate hospital/hospice based practice in the community due to lack of same structure and resources in community. Solo practice	**30**
Pype et al. (b) (2014) [26] Belgium	To explore General practitioners and PHCT nurses learn during collaborative practice? To explore General practitioners and PHCT nurses learn during collaborative practice? To explore General practitioners and PHCT nurses learn during collaborative practice?	267General practitioners 73nurses	Survey	**Motivation to learn:** Experienced general practitioners preferred learning. Attrition of knowledge and skills. The science of palliative care as ever evolving warranting continuous upgradation. **Learning needs:** General practitioners preferred to learn about physical and psychological symptom management. Other topics such as religion and spirituality, teamwork and organisational items were preferred. **Learning style:** General practitioners preferred discussion and reflection followed by observation and didactic learning. They preferred work place learning, felt comfortable learning from palliative home team nurse/community nurse followed by patients/family and GP colleagues. They preferred learning by listening and observing for topics such as teamwork building, religious and psychosocial topics) and by discussion and reflection on psychosocial topics. **Facilitators of learning**: General practitioners learnt from patients followed by nurses, [referred learning from mentor, peers and general practitioner colleagues. Feedbacks and learning from mistakes were preferred by some as this needed a high degree of trust between team members. **Barriers for learning**: Solo practice	**31**
Hermann et al. (2019) [38] Australia	To explore Australian General practitioners’ perceptions of barriers and enablers to the provision of palliative care.	25General practitioners	Inductive content analysis	**Motivations to learn**: Felt palliative care as central to caring, emotional attachment with patients and family, sense of responsibility towards their patients/family. **Learning needs**: Need for training as to when to initiate EOLC, how to discuss treatment when patient/family are in denial, lack of knowledge on available resources for palliative care guidelines especially not knowing symptom management and when to stop drugs that were once therapeutic, resources for training, accessibility to specialist palliative care team. Need for debriefing in case of emotionally draining situation in practice. **Learning styles**: They “gained by doing”and felt integrating training in routine clinical practice under the mentorship of experienced general practitioner/specialist palliative care team. They felt this could be done through small group problem based discussion at their practice site; locally available resources. Online courses benefited rural GPs. **Facilitators of training**: General practitioners reported that education programs should involve repeated information sessions, during and out of business hours, with additional follow-ups to allow for further discussion, promoting Palliative care as a sub-speciality in general practice. **Barriers for learning:** Palliative care as time consuming, palliative care as being complex and emotionally burdensome, lack for clearly defined roles and responsibilities, lack of recognition/appreciation of their work by specialists, mistrust in their service provision by specialist and patients/family, poor communication from specialists about the intent of treatment, poor remuneration, lack of knowledge about the available opportunities for training, lack of standardisation and accreditation of palliative care.	**30**
O’Connor and Le-Steere [39] (2006) Australia	To explore General practitioners’ attitudes to palliative care in a rural center of Western Australia To understand factors contribute to General practitioners attitudes to palliative care in a rural center of Western Australia To explore the perceived barriers to the provision of palliative care in a rural center of Western Australia	10General practitioners	In-depth interview Qualitative data analysis	**Motivation for learning**: General practitioners felt palliative care and pain relief was their core responsibility; their guilt and remorse of not having been able to alleviate symptoms in end-of-life care; their need to support patients who they had cared for a prolonged period of time and developed an emotional bond with. **Learning needs**: General practitioners expressed the need for training in pain and symptom management followed by communication skills training as they dealt frequently and intimately with patients/family. They expressed the need for enhancement of skills, tacts, diplomacy in dealing with delicate situation with patient and family. They felt the need for training in dealing with spiritual issues of patients and psychological issues such as depression and emotional problems. They expressed the need for communication skills training in case of denial or bargaining, learning self- care and coping with the situation especially when caring for dying younger patients whom they had known for long, general practitioners with young off-springs of similar age as their dying patient. Need to deal with family’s denial about terminal illness, resolution of conflict and supporting family’s with anticipatory grief. **Learning style**: General practitioners felt a need for team approach in palliative care as it provided emotional support, and aided in time and management plans. Telephonic or web based training may be a better option for rural practitioners. **Barriers for learning:** General practitioners might have less consistent support system, may be temporary at work due to family and personal commitments, or lack time due to work pressure. Palliative care can be emotionally draining and depressing, loss of control on their patients with palliative care becoming a specialised field, perception of no newer developments in palliative care.	**30**
O’Connor and Breen [40] (2014) Australia	The study aimed to explore General practitioners’ understandings of bereavement support and their educational and professional developmental needs in relation to providing bereavement care	19General practitioners	In-depth interview using social constructionism approach	**Motivation for learning**: Being human to patient concerns, personal experience of loss and personal grieving process, long standing relationship with patient and bereft family, sense of responsibility to support bereaved family. **Learning need**: General practitioners felt ill-equipped with counselling family with complicated grief, were not able to distinguish between grief reaction and depression. **Learning style**: Minority of General practitioners preferred ‘on the job’ experiential learning over CPD or self-learning. They felt learning the theoretical principles of grief counselling, applying them in practice and learning from patient feedback for betterment in the service. **Barriers for learning**: Challenges of learning from specialist psychologists/psychiatrists (with incomplete reporting by the latter), unawareness of local resources for counselling.	**30**
Assing Hvidt et al. (2016) [41] Denmark	What were the points of agreement and disagreements among General practitioners in Denmark concerning how the existential dimension is understood, and when and how it is integrated in general practice?	31 General practitioners General practice setting	Hermeneutic-phenomenological research methodology	**Motivation to learn**: General practitioners felt the need for whole person care and emphasised the importance of addressing suffering as integral to health and illness. **Learning needs:** They felt the need for systematisation and standardisation in providing spiritual care, challenged as to not knowing the right moment to refer their patient to chaplain. **Learning style: They** felt that spirituality is intuitive and it grows over years of practice and relationship with patient. **Barriers to learning**: They had apprehensions about discussing death and dying as they felt it would mean causing discomfort to the patient or perceived as taboo, felt a lack of specialist competence in addressing spirituality as they felt they were treading an unknown territory, They feared intruding into patient’s privacy by broaching the topic of spirituality	**27**
Becker et al. (2010) [42] Austria	To identify the preferences of the General practitioners’ and nurses’ regarding the specific design of training seminars in palliative care To gain insight into which educational topics, timeframe, location and group designs are likely to attract a majority of different professional groups	897 General practitioners 933 Nurses	Survey	**Learning needs**: General practitioners felt the need for training in pain and symptom management followed by handling psychosocial and ethical issues and coping strategies **Facilitators of training**: General practitioners especially rural general practitioners preferred evening courses and weekend courses, were to pay for their training, willing to travel for training (including traveling abroad), preferred a mentors and peers from a multidisciplinary team for training	**26**
Straatman and Miller (2013) [43] Canada	To assess the experience with and confidence of practicing family/GP and paediatricians in providing paediatric palliative care	43General practitioners 56General paediatricians 14pediatric subspeciality	Survey	**Motivation to learn**: Rarity of the life limiting illness, complexity of illness and prolonged care, current medical knowledge and experience as inadequate to meet the needs of paediatric palliative care patients, **Learning needs**: Management of other symptoms followed by management of pain and nutrition, self-care and care of the team, training in spirituality to cater to self and the team, debriefing with colleagues and teamwork. **Learning style: They** preferred remote learning such as internet based or correspondence learning followed by workshops. **Facilitators of learning**: They preferred to attend workshops over weekends	**24**
Slort et al. (2011) [44] Netherlands	(1) To understand facilitators for GP–patient communication in palliative care are reported by General practitioners (2) To understand the barriers for GP–patient communication in palliative care are reported by General practitioners	20 GENERAL PRACTITIONERS	Focus group discussion Qualitative data analysis	**Motivation for learning**: Willingness to provide palliative care, positive attitude towards helping their patients, paying regular home visits; honouring the patient’s dignity, autonomy, wishes, and expectations; ensuring continuity of care; longstanding GP–patient relationship. **Learning needs**: They expressed difficulty in dealing with patient’s fears and emotional distress. Need for training in handling a troublesome relationship with the patient and conflicts within the family. Communications skills training as aide to elicit patient’s wishes and expectations. They felt ill equipped in controlling their patient’s symptoms adequately	**25**
Taubert et al. (2011) [45] Cardiff, UK	To explore problems relating to palliative care and symptom control in out of hours setting	9 General practitioners	Interpretative Phenomenological analysis	**Motivation to learn: G**eneral practitioners feared treading the unknown territory of end-of-life care **Learning needs**: Gaps in knowledge or skills forgotten especially in out-of-hours care, lack of knowledge appeared to have an effect on their prescribing habits, perhaps making them less inclined to increase a patient’s drug dose. Due to lack of continuity in out-of-hours care they lacked confidence on prescribing and escalating dose of opioids. **Learning style**: They preferred problem-based learning by following up on their patients, reflective learning so to learn from mistakes/non- success, referring to internet and textbooks for learning palliative care. Some felt the need for previous experience for a better performance. Some even felt exposure to hands on training in palliative care was beneficial in end-of-life care. **Barriers of learning**: They felt insecure to broach the topic on lack of knowledge, out-of-hours practice and shift jobs gave them minimum opportunity to reflect on their practice, most feared harming the patient or being medico-legally recriminated. Most training occurred “in-hour” which did not help out-of-hours care.	**25**
Rhee et al. (2008) [46] Australia	Study aimed to determine the level of participation of Australian urban General practitioners in palliative care, and to determine the main barriers facing them in providing this care.	269General practitioners	Survey	**Motivation for learning**: Strong emotional bond with patients, long years of practice, more clientele, more older and sicker patients motivated learning. **Learning needs**: Needed training in use of syringe drivers, providing psychosocial care or complex symptoms in terminal care. **Learning styles**: General practitioners preferred workshops followed by written material and online learning **Barriers for learning**: Lack of time due to work pressure followed by home visits, family/personal commitment, younger general practitioners, poor remuneration, demoralised when specialists take over the care	**28**
Rhee et al. (2018) [47] Australia	Study explored the views on the role that General practitioners should play in the planning and provision of end of life care in Australia and Important barriers and facilitators to their involvement including suggestions on what could be changed or improved.	11General practitioners 10SPCC	In-depth interview	**Motivation for learning**: General practitioners who had inclination to provide end-of-life care and advance care planning, supported the patients/family for a prolonged period of time, reciprocation and appreciation from family of feeling supported, long term relationship with patient and family, sense of responsibility towards patient and community. **Learning style:** General practitioners wanted real-life exposure to gain confidence in skills. They felt the need for working in collaboration with specialist palliative care, easy access to specialists and ongoing communication with specialists. They desired training under an experienced General practitioners or in a palliative care centre. **Barriers for learning**: General practitioners catering to younger population had less sick patients who may not follow up to build a long term relationship which may challenge end-of-life care discussion in this population that resulted in less involvement in palliative care	**25**
Meijler et al. (2005) [48] Netherlands	A study to explore concerns of GENERAL PRACTITIONERS in relation their educational needs in palliative care	40General practitioners	Focus group discussion Qualitative data analysis	**Motivational factors for learning**: Palliative care as a valuable part of their care. Being trained in palliative care makes one a good physician. **Learning needs**: Pain was one of the most difficult problems especially certain specific aspects of pain management such as “when” and “how” to start opioids, challenges of subcutaneous administration of opioids (use of combination of medications), effects and side effects of nerve blocks, Other issues included use of parenteral nutrition/PEG feeding, diagnosing and management of delirium with special emphasis on subcutaneous midazolam administration, depression/insomnia and difficulty in distinguishing adjustment disorder and sadness, Insomnia as an area of concern as General practitioners found challenge in distinguishing the cause as delirium, fear of dying, or anxiety. Communication was frequently mentioned as being complex especially when patients transitioned from curative to palliative phase of treatment, addressing caregiver concerns, need for training in conflict resolution and denial especially the use of right attitude in dealing with these issues They expressed the need for debriefing sessions and understanding of “how to develop and discuss” and share ethical aspects of care with other professionals. **Learning style**: They felt the need to learn to reduce complex problems to solvable solution through problem-based education. **Barriers for learning**: Non recognition of their role, lack of clarity in roles and responsibilities, care as time consuming, complexity of care, land up spending more time to compensate for the helplessness led to neglect of other patients, feared medico-legal recrimination.	**25**
Junger et al. (2010) [49] Germany	To examine potential barriers, incentives, and the professional self-image of general paediatricians with regard to paediatric palliative care	Pediatricians in general practice	Sequential exploratory methods: Qualitative indepth interview- 5pediatricians Quantitative method using survey methods-293	**Motivational factor for learning**: To help the child as a person rather than a diseased, experience in clinical practice as being essential component of care, Intuition that they will be able to extend the care of the dying child, Trusted key person who accompanied the family for many years. **Learning needs**: Most lacked knowledge as to what diseases are included in palliative care, decision-making in children with life limiting illness can be complicated thus had challenges of integrating palliative care, underreporting of pain and fear of side effects of opioids, apprehensions about discussing death and dying with patients/family, need for coping and self-care, debriefing and mutual support. **Preferred learning style**: Seminars were most frequently mentioned followed by self-study and lectures. **Barrier for learning:** Felt vulnerable discussing on transition to end-of-life, emotional burden of caring for a dying child, poor support by specialist, solo practice, unawareness of specialist palliative care team, fear of medico-legal recrimination	**27**
Magee and Koffman (2016) [50] UK	To examine the perceived confidence of OOH General practitioners in symptom control, end of life prescribing, communication skills, and to identify their educational needs and preferences.	203 General practitioners	Survey	**Learning needs**: Educational preference was closely linked to low confidence in palliative care emergencies, symptom control in non-cancer palliative care and use of syringe driver. Frequently requested training in end-of-life care pathways, opioid prescribing, management of breathlessness, agitation/confusion. **Learning style**: E-learning was the preferred style followed by workshops, Interactive case-based discussion, learning on the job and didactic lectures. **Facilitators of training**: Workshops with multiprofessional lecturers and other General practitioners participants	**28**
Barcley et al. (2003) [51] Wales, UK	To investigate the training in palliative medicine of General practitioners throughout Wales during their careers.	590 General practitioners Mixed setting	Survey	**Motivation to learn**: More experienced as a general practitioner, past experience in home care and care of the dying had a higher predilection for training in end-of-life care. Continuity of care as a central role of general practitioner. **Learning needs**: They preferred training in pain and symptom management, use of syringe drivers, bereavement care.	**22**
MA Wakefield et al. (1993) [52] Australia	To assess Australian General practitioners assessment on opinions and management practices in palliative care for terminally ill patients	108 General practitioners	Survey	**Learning needs**: General practitioners felt a lack of competence in managing psychosocial issues of terminally ill patients and dealing with emotional distress of the relatives, felt incompetent in managing pain, had anxieties about use of opioids and side effects of opioids, management of tolerance to opioids, management of other symptoms such as hypoxia and insomnia, training in communication skills with dying patients, and bereavement counselling. **Facilitators of training:** Felt the need for training under the mentorship of specialist palliative care team	**20**
Shipman et al. (2001) [53] London UK	The study explored the General practitioners’ educational preferences in palliative care, focusing particularly on variations in preferences by location of practice (inner-city, urban and rural General practitioners)	640 General practitioners	Survey	**Motivation for learning**: General practitioners who were more confident in prescribing analgesia were more likely to prefer further training. **Learning needs**: They needed training in symptom control for non-cancer patients due to prolonged period of care they provided to non-malignant patients. They wanted training in use of analgesics, syringe driver, nausea/vomiting management, counselling skills, communication skills and breaking bad news. **Barriers of learning**: General practitioners perceived barrier in training as most trainings were oncology focussed which did not help in managing non-malignant palliative care which comprised major part of their practice	**23**
Shipman et al. (2002) [54] London UK	To understand the General practitioners use of and attitude towards specialist palliative care in different geographical context	49General practitioners IDI 8GENERAL PRACTITIONERS one FGD	Indepth interview and FGD Qualitative data analysis	**Motivation to learn**: General practitioners learnt from specialists only if their preference were in alignment with those of the specialist’s **Learning style**: They contacted specialists for support through telephone/face-face for updating their knowledge on a case to case basis, used the hospice symptom control book for updating knowledge, they contacted specialists for guidance on symptom control and emotional support, some attended courses in hospice or palliative care centres. **Facilitators of learning**: General practitioners involved themselves in joint care with specialists if the encounter was amicable. **Barriers of learning**: Low incidence of palliative care cases in practice may inhibit learning. They kept away from learning from specialists when they felt uncomfortable working together or when they feared accusation, had past bitter experience or feared conflict in care	**20**
Samaroo (1993) [55] Canada	The survey was conducted to identify physicians’ and nurses’ perceived educational needs related to death and dying et al.	102General practitioners 263RN	Survey	**Learning needs**: Pain, dyspnoea, restlessness and confusion were most discomforting for the physicians followed by anger and demanding behaviour of patients/family. Withdrawal avoidance were most discomforting with regards emotional/behavioural component. **Learning style**: Physicians desired specialized training with the hospital based palliative care team **Facilitators of training**: they preferred quarterly topical in-services, self-learning modules, extension courses for credit, and one-day on-site programs, quarterly case rounds, and half-day workshops.	**19**
Lloyd-Williams et al. (2006) [56] North Wales, UK	A study to evaluate palliative care provision and training needs of general practitioner in rural areas of North Wales	94General practitioners	Survey	**Motivations for learning**: Younger General practitioners had keen interest in palliative care; felt palliative care as a part of their care provision; felt central to coordinating palliative care for patients; relationship with family and longer duration of this association with patients and family triggered the need to enhance their skills. **Learning needs**: They felt the need for training in pain and symptom management followed by dose titration of opioids, breaking bad news, psychosocial needs and bereavement care. **Learning style**: General practitioners felt the need for experiential learning over didactic lectures; placements within hospice or palliative care team as beneficial as observing and discussing difficult cases helped better uptake of knowledge. **Facilitators of training**: General practitioners preferred evening meetings. They appreciated learning from peers such as colleagues, palliative care team, and palliative care nurse. **Barriers for learning**: Solo practice, lack of support system, Time constraint due to busy practice	**20**
Jhonston et al. (2001) [57] Northern Ireland	To carry out an educational needs assessment in palliative care of general practitioners and community nurses.	611 General practitioners	Survey	**Motivation for learning:** General practitioners were motivated by their current palliative care practice, and felt that palliative care was a core element of their practice. **Learning needs**: Symptom management other than pain such as fatigue, anorexia, unpleasant smell, anxiety and depression followed by pain management, use of syringe driver, bereavement care and dealing with complexities of spiritual care. **Learning styles**: Order of preference: lectures by specialists, case discussion with specialists, experiential learning in hospice, research and audit as need for reflection on practice and self-learning through computer and information material. **Facilitators of learning:** Preferred a multidisciplinary learning by multidisciplinary team; interactive learning and video-feedback. **Barriers in learning**: lack of locally based courses, lack of dedicated time and the expense of providing locums or of self-funding courses.	**23**

#### Review themes

Five themes were generated in the review. These themes were: motivation for end-of-life care learning, end-of-life care learning needs, preference for a learning style, perceived facilitators of learning, and perceived barriers to learning. Refer to Additional files 4 and 5 for the table narrating the themes and thematic diagram.

#### Theme 1: Motivation for end-of-life care learning

Motivation is defined as an intrinsic trigger that allows for sustained goal-directed activity [59, 60]. Self-directed learning enhances the learner’s competence, and belongingness facilitates motivation [61, 62]. Perceptions of value addition, peer recognition, previous learning experiences, and individual goals all trigger learning [63]. The need to provide palliative care, self-actualisation needs, relevance to practice, sense of responsibility, and therapeutic bond motivated them to learn about end-of-life care.

GPs are internally motivated to enhance their capacity to alleviate the suffering of their patients on the pretext of achieving their self-actualization needs [39, 40, 44, 57]. Also, having a larger clientele of geriatric and sicker patients piqued their interest in end-of-life care learning [44, 46, 49, 51, 57]. Unmet self-actualisation needs included a desire to integrate palliative care into routine care [38, 48, 49, 56], a perceived need for self-transformation [48], coping with own bereavement [39], and a perceived inability to manage symptoms [40], all of which instilled a sense of powerlessness, helplessness, and emotional burden of caring [24, 38–40, 48] for which they needed a recourse [48].GPs accessed training only if the training [24, 41, 54] and the trainer’s skills were in alignment with their needs [54] and helped address complex end-of-life care needs [24, 26]. A therapeutic bond developed with their patients over a prolonged period of caring [47, 51, 53], at various stages of their illness [38, 39, 46, 47, 49], instilled a sense of responsibility towards their patients [25, 39, 40, 49]. They believed in helping a patient as a whole person and not as a disease entity [49] and felt the need to address the complex sufferings of patients while honouring their dignity, expectations, and wishes [41, 44].

#### Theme 2: End-of-life care learning needs

Human beings encounter newer experiences in their journey of life that helps them evolve [64]. Some of these experiences could be in synchrony with their past practices while some could be conflicting [65]. They tackle the unrest arising out of these conflicting thoughts in three ways: by preserving the existing schema and ignoring the new contradictory knowledge; by preserving both the contradictory knowledge and appropriating it as the situation requires; or by constructing a new concept that aids in resolving the contradictory knowledge [65]. Following were the learning needs: accessing palliative care, learning about pain and symptom management, communication skills, compassionate care, addressing the caregiver’s needs, the ethical and medico-legal aspects of end-of-life care, and teamwork.

GPs expressed the need for a framework for accessing local specialist palliative care resources [24, 38, 39, 47, 49], guidelines on out-of-hours care [24], timing and criteria for referral to specialist services [40, 47] and knowledge of a multidisciplinary team approach [24, 39]. Among physical and psychosocial educational needs, pain management [39, 42, 43, 48, 50, 52, 55] and psychosocial issues [26, 39, 46, 52, 56] were perceived as most important aspects of learning followed by fear of death and dying [39, 44, 48, 57], anger and demanding behaviour [55], nutrition [43, 48], spiritual needs [25, 43, 57], and religious concerns [25]. GPs expressed the need for training in communication skills and breaking bad news that enabled them to make decisions on chronic illnesses [49] and facilitate transitions to end-of-life [48, 50]. They appreciated training that might enable them to have the right attitude, diplomacy, and skills [39, 48] to resolve conflict [39, 44, 48], denial, and bargaining [38, 39, 48] and handle vulnerability around death and dying [49]. They expressed the need for training in handling the emotional distress of family caregivers [48, 52], and anticipatory grief [39], and bereavement support [49, 52, 56, 57]. GPs felt a need for guidelines to enable them to provide care in the best interests of their patients [24, 44] and techniques to discuss ethical and medico-legal aspects of care with their patients, families and colleagues [47, 48]. They expressed the need for skills in developing a team and team work, garnering mutual support from colleagues in a multidisciplinary team [43, 49] and for debriefing sessions to cope with emotionally burdensome situations [38, 43, 49].

#### Theme 3: Preference for a learning style

A learning style is defined as a set of “characteristic cognitive, affective, and psychosocial behaviours that serve as relatively stable indicators of how learners perceive, interact with, and respond to the learning environment” [66]. When individuals become aware of their strengths and weaknesses, they become motivated to learn and become lifelong learners [67]. It may be essential to match learning styles with learning preferences and tailor the style to the content and context of learning in order to enhance learning [68–70]. GPs preferred learning styles that were experiential, pragmatist, self-directed, and didactic.

According to GPs, expertise grows with experience [24, 25, 39, 41, 56] and through interaction with patients/families [24–26, 41]. They preferred work-based learning under a mentor [24, 26, 38, 40] or co-management with a palliative care team [24, 47]. They wished to reflect on their practice under a mentor [25, 26, 40, 45, 49, 50] either through virtual interaction [24, 54], by the patient’s bedside [47, 55, 57], or through audited data [57]. They also expressed the need for problem-based learning by patient follow-up and learning from mistakes [45, 48]. GPs preferred self-directed learning through web-based or printed resources [26, 38–40, 43, 45, 46, 49, 50, 55]. Some of them were computer-based learning [57], correspondence learning [4], guidelines-based learning [54, 55], E-learning [24] and learning by researching [57]. Other forms of learning included lectures [49, 56, 57] and workshops on team building and addressing religious and psychological needs [25, 40].

#### Theme 4: Perceived facilitators of learning

Facilitation of learning is an essential process that empowers learners to evolve in the learning process through self-evaluation and interchange [71]. According to Brockett, attending, responding, and understanding are important for effective facilitation, which builds a foundation for a constructive and meaningful learning environment [71]. An amicable learning environment, mentorship, appropriate timing, and feedback facilitated end-of-life care learning.

GPs felt the need for an amicable, safe, non-intimidating, non-judgmental, trustworthy and respectful learning environment where their deficiencies were not exposed to their patients [25, 26]. Mentorship is a bidirectional process [72] and provides a safe space for the learners to interact [73]. GPs expressed the need for mentors from a multidisciplinary team [25, 48, 50, 56] and a small group [25, 38] interactive session with multidisciplinary peers [42, 50]. Due to time constraints, solo practice, remote practice, and multiple concurrent responsibilities, GPs are unable to attend educational programs during office hours [74, 75]. GPs felt that learning was a lifelong process [25, 38] and preferred to attend half-day workshops [54], quarterly topical in-service training [55] and quarterly case rounds [55], courses out-of-business hours [38, 42] over the weekend [42, 43]. Goal-directed feedback keeps the learner motivated and engaged in order to strike a balance between one’s goals and the expectations that one can achieve these goals [76]. GPs preferred feedback from specialists and peers in the team [25] and patients and families if it was completed in a time bound manner [24, 40]. They preferred to avoid feedback from dying patients or their families, as this might cause discomfort to the respondents [24].

#### Theme 5: Perceived barriers to learning

The learner’s decision to participate in learning is influenced by constraining factors that could be dispositional, situational, institutional, or academic in nature [77, 78]. End-of-life care learning was hampered by a lack of resources, individual and professional challenges, disempowerment, conflicts, and training that was not aligned with clinical practice.

GPs had apprehensions about initiating discussions around end-of-life care as this was perceived as taboo by their patients and families [41, 49]. They feared treading into their patients’ or family’s private spiritual space [41] and perceived a lack of competency in addressing end-of-life care concerns [41]. Other limitations included fear of medico-legal recrimination [45, 48] and the emotional burden of care [39]. GPs felt a sense of insecurity when broaching their deficiencies in knowledge [45]. Younger GPs with fewer years of experience had less autonomy to focus on palliative care as a choice of specialty [41, 47, 51]. Some felt they had enough knowledge to address their patients’ palliative care concerns [54] and that there were no new developments in the field that warranted up-gradation of knowledge [39, 40]. Additionally, family and personal commitments gave them less time for palliative care provision [46]. GPs who had solo practice or practiced in remote areas had fewer support systems that precluded access to training [49, 51, 53]. Excess work pressure [41, 54, 57] with the resultant lack of time [24, 25, 38, 39, 46, 48, 54, 57], poor remuneration [38, 46], and the temporary nature of the job [39] were hindrances to training. They were also limited in their access to training programs because they had to self-fund their courses or provide compensation to organise locum GPs in their absence [57]. When specialists took over care [24, 25, 46], GPs felt a loss of control over their patients [39]. They were disillusioned when specialists or patients lacked trust in them [24], or when they were not acknowledged [38, 48]. Lack of clarity in roles and responsibilities led to conflict in care coordination [25, 40, 54]. GPs were apprehensive about confronting specialists for fear of being reprimanded [54]. Furthermore, previous negative experiences and delays in receiving responses from specialists limited future interactions with specialists [2]. Most end-of-life care training programs focused on oncology, providing fewer opportunities for them to learn non-malignant end-of-life care skills, which accounted for a significant portion of their practice [53]. Training occurred mostly during office hours in the hospital setting [45, 57] and skills gained could not be replicated in the community due to differences in the infrastructure in both settings [25].

## Discussion of the review findings

### Social constructivist learning theory as a framework for interpreting the results

Learning is an internal cognitive progressive process [61]. Learning is contextualised in a lived situation in which the learner constructs meaning to make sense of one’s past beliefs and practices [61, 79]. Social constructivist learning theory enables us to theorise about how individuals attribute value to their learning within a social, emotional, temporal, and cultural context [80, 81]. In this review, the theory was used as a lens to explore GPs’ insights on end-of-life care learning and what processes do they adopt to acquire and construct knowledge in end-of-life care [81].

The therapeutic bond associated with long-term care [82] uniquely positions GPs to address patients’ physical and situational challenges at the end-of-life [83, 84]. GPs expressed a lack of competency in the management of pain and other symptoms and communication skills, which reflected a major gap in the holistic approach to end-of-life care. Their perceived lack of access to the palliative care teams exacerbated their lack of competency [85], which was further compounded by an ambiguous division of roles and responsibilities [86].

Individuals’ progressive experiences sometimes foster contradictions to their present understanding or beliefs, making them insufficient and thus leading to dissonance in the cognitive structure [61]. GPs bring their life and clinical practice experience to the learning environment [61]. Their past experience is put into question when they encounter new knowledge [61]. However, the dissonant thought arising out of this conflicting knowledge will be resolved only if the learner feels empowered [61]. A competency-based trust is the trustor’s belief in the trustee’s ability to complete a task [87]. The assured mutual support and acknowledgement of the specialists motivated GPs to invest in learning, and the contrary led to dissatisfaction and demoralisation. Distrust could undermine one’s self-esteem by questioning one’s capability [88], fracturing interpersonal collaboration [87], and demoralising the individual from task-performance and accessing learning [88].

Learners strive to preserve their knowledge schema [89] and shape the new experience to conform to this schema [6]. In order to reduce the uncomfortable feelings of dissonance arising out of conflicting encounter, they will engineer ways to remove the dissonant cognition [7] by transforming the knowledge structure to accommodate the new knowledge [6]. However, the transformation in the schema will only occur if the history of the experience is honoured and its future is appreciated [6] or if the learner perceives this as valuable information to address the gap in their knowledge [7]. As the evidence base in end-of-life care is ever evolving, there is a constant need for updation in the knowledge of GPs and they access training programs in end-of-life care to resolve the conflicts in their daily practice [41, 88, 90]. Diverse clinical practice scenarios trigger learning [91] through reflective identification of knowledge gaps in the quality of care [92]. In the process of learning, GPs gain multiple perspectives by interacting with mentors and peers in the learning environment or at work [6, 61]. They critically reflect on multiple perspectives, amend their practice, and reflect and consolidate them [6, 61, 93].

Learners constantly acquire and apply knowledge under the guise of autonomously developing, maturing, and enhancing themselves [61] and preserving their identity [85]. GPs in this review felt accountable for the care they provided. They also reported end-of-life care as self-actualising, as it helped them gain inner satisfaction of having embraced their patients’ concerns and addressing them [61].

Learning preference has been considered in this review to include a broader set of factors such as learning styles and intrinsic and extrinsic environmental factors that influence learning, including when and how learners prefer to pursue learning [94]. Although traditional didactic learning styles are presumed to be prescriptive in nature, when the learning occurs in a small group interactive session, it helps to bring an attitudinal shift, integrate multiple thoughts, and lead to better understanding [95]. Similarly, online or e-learning in the current era has cut across geographical barriers. With either the information on web-pages or through online training, learners have the opportunity to construct meaning through active participation and self-directed inquiry [96].

Learning is a self-directed process [61] and learners are provided ample space to democratically express their intellectual and emotional content [61, 89]. Language mediates this expression and helps learners interact with others in the learning environment, probe each other’s thoughts, and understand the way each individual interprets reality [85]. Learners then negotiate, defend their positions, and create meaning on their own terms [89]. Teacher acts as a scaffolding agent, facilitates the interchange within the group, and gives an apportioned degree of emphasis on the contents [61, 85]. The GPs in this review valued experiential and reflective learning under the mentorship of a specialist as it gave them the opportunity to make amends in their practice.

Feedback is an integral component of learning and helps improvise practice. Feedback is a mechanism that improves the thought process of the learner in order for the learner to accurately address the problem at hand, thus enhancing both learning and performance [74]. It can reduce the uncertainties arising from the gap between the current and desired levels of performance [97]. GPs in the review realised the importance of addressing this gap by receiving feedback from specialists and patients/families. However, the studies in the review emphasised the importance of a trustful learning environment, the timing of feedback, and the person who recorded the feedback as having an impact on performance and future learning [98].

### Critical reflection on other learning theories that may inform social constructivist learning theory

As per the cognitivist and constructivist theories, GPs explore knowledge with the pretext of enhancing their expertise in delivering end-of-life care [63]. When faced with a conflicting situation in clinical practice, they seek information from multiple resources, decide on the best possible solution, and integrate the new knowledge on their own terms [63]. Bandura’s social cognitive theory extends this theory by including a social and cultural milieu in learning [63]. GPs learn from experienced GP colleagues or palliative care specialists in multidisciplinary team meetings or case discussions [24, 25, 47] in order to draw consensus on the mutually derived path to knowledge and practice [88]. In this way, GPs receive a legitimate endorsement of their practice [88]. Individuals self-regulate their learning, and the learning is influenced by internal and external environmental factors which may facilitate or impede learning [63]. According to Vygotsky, for learning to occur, it has to be within the zone of proximal development [63]. In a conflicting situation, GPs adopt one of the two paths to learning; either they reject the new experience as trivial or they incorporate the new experience into their knowledge schema [6, 7]. Connectivism is yet another theory where learning happens beyond human interactions [99]. GPs connect to their learning environment through human and non-human appliances such as the internet and webpages and thus construct knowledge based on the best possible answer to plural opinions [96].

### Limitations and strengths

The search strategies were limited to studies published in English. Some studies explored the learning needs of a mixed population of GPs and nurses involved in general practice. However, the learning needs were clearly disaggregated from one another. Three studies [42, 50, 55] had a poor response rate and five papers had a response rate greater than 50% [25, 46, 51, 53, 57]. Although some studies had a poor response rate, they had a significant number of participants who gave their perception of the learning needs in end-of-life care.

Although the studies included in the review were dated from 1993 to 2019, most studies were published in the last 10 years. The learning preferences of the most recent generation of GPs were not very different from those of their predecessors. Two reviewers independently reviewed the articles, compared the search results, identified the methodological rigour using Hawker’s tool, discussed the discrepancies in the findings, and resolved the discrepancies by mutual consultation with the help of the third reviewer. The results of the review were able to generate themes that were able to satisfactorily answer the review question. Theoretical positions might have influenced theme generation and themes might align closely with the theory. Since we were aiming at exploring multiple perspectives of the GPs, the heterogeneity of the articles, the combination of quantitative and qualitative methods employed, and data obtained from across the continents, increased the depth of our understanding, allowing it to be replicated in different settings.

### Future considerations

Four studies in the review revealed that GPs having solo practice had limited access to end-of-life care training [39, 49, 51, 53]. Thus, knowing their learning needs will give a different perspective on factors that may influence their learning. All the studies included in this review were conducted in Europe, Australia, and Canada [39, 49, 51, 53] with no insight from other geographic locations. Moreover, patients’/caregivers’, or palliative care team’s views on the care provided by GPs will further bridge the gaps in end-of-life care provision.

### Implications for policy and practice

Patient-related suffering and how empowered GPs feel in addressing them determine their educational needs in end-of-life care. Thus, identifying the challenges that GPs face in physical and psychosocial care, communication skills and coordination/collaboration of care will add relevance to the training program. There is need for a paradigm shift in training programs from a formal training to a more experiential and reflective learning under mentorship in order for training programs to be sustainable over long run. Additionally, every training programs must have an inbuilt feedback and evaluation mechanism as this will ensure sustainability and help GPs amend clinical practice on an ongoing basis.

## Conclusion

The review suggests that GPs’ preference for learning end-of-life care was influenced by the value that they ascribed to their learning. GPs who had a sense of responsibility or a therapeutic bond with their patients were motivated to undergo training in end-of-life care. Diffidence in providing end-of-life care, challenges on a personal and professional front, and a feeling of disempowerment inhibited them from accessing training. GPs preferred experiential and reflective learning, under the guidance of specialists. They perceived feedback as an integral component of learning and performance. They preferred feedback if it was provided sensitively and in a respectful, non-intimidating, and trustworthy environment.

## Supplementary Information

**Additional file 1.** Search Terms

**Additional file 2.** List of hand searched journals

**Additional file 3.** Quality Assessment of the studies using Hawker’s tool

**Additional file 4.** Systematic review themes and subthemes

**Additional file 5.** Thematic diagram of General Practitioner’s End-of-Life Care learning preferences

**Additional file 6.** Hawker’s tool

## Data Availability

All data generated or analysed during this study are included in this published article and its Supplementary information files.

## References

[1] Mitchell GK (2002). How well do general practitioners deliver palliative care? A systematic review. Palliat Med.

[2] Mitchell GK, Reymond EJ, McGrath BP (2004). Palliative care: promoting general practice participation. Med J Aust.

[3] Murray SA, Boyd K, Sheikh A, Thomas K, Higginson IJ (2004). Developing primary palliative care. BMJ..

[4] Forrest S, Barclay S (2007). Palliative care: a task for everyone. Br J Gen Pract.

[5] Gott M, Seymour J, Ingleton C, Gardiner C, Bellamy G (2012). 'That's part of everybody's job': the perspectives of health care staff in England and New Zealand on the meaning and remit of palliative care. Palliat Med.

[6] Kegan R, Illeris K (2009). What “form” transforms? A constructive-developmental approach to transformative learning. Contemporary theory of learning.

[7] Harmon-Jones E, Mills J, Harmon-Jones E (2019). An introduction to cognitive dissonance theory and an overview of current perspectives on the theory. Cognitive dissonance: re-examining a pivotal theory in psychology.

[8] Stern C, Jordan Z, McArthur A (2014). Developing the review question and inclusion criteria. Am J Nurs.

[9] Charmaz K (2014). Constructing grounded theory.

[10] Carr SM (2005). Knowing nursing - the challenge of articulating knowing in practice. Nurse Educ Pract.

[11] Jones MG, Brader-Araje L (2002). The impact of constructivism on education: language, discourse, and meaning. Am Commun J.

[12] Jonnaert P, Masciotra D, Barrette J, Morel D, Mane Y (2007). From competence in the curriculum to competence in action. Prospects..

[13] Burnham JF (2006). Scopus database: a review. Biomed Digit Libr.

[14] Carey ML, Zucca AC, Freund MA, Bryant J, Herrmann A, Roberts BJ (2019). Systematic review of barriers and enablers to the delivery of palliative care by primary care practitioners. Palliat Med.

[15] Rhee JJ, Grant M, Senior H, et al. Facilitators and barriers to general practitioner and general practice nurse participation in end-of-life care: systematic review [published online ahead of print, 2020 Jun 19]. BMJ Support Palliat Care. 2020:bmjspcare-2019-002109. 10.1136/bmjspcare-2019-002109. 10.1136/bmjspcare-2019-002109 32561549

[16] Greenfield K, Holley S, Schoth DE (2020). A mixed-methods systematic review and meta-analysis of barriers and facilitators to paediatric symptom management at end of life. Palliat Med.

[17] Mitchell G, Aubin M, Senior H, et al. General practice nurses and physicians and end of life: a systematic review of models of care [published online ahead of print, 2020 Jul 27]. BMJ Support Palliat Care. 2020:bmjspcare-2019-002114. 10.1136/bmjspcare-2019-002114. 10.1136/bmjspcare-2019-002114 32718955

[18] Kelley LT, Coderre-Ball AM, Dalgarno N, McKeown S, Egan R (2020). Continuing professional development for primary care providers in palliative and end-of-life care: A systematic review. J Palliat Med.

[19] Mitchell GK, Senior HE, Johnson CE (2018). Systematic review of general practice end-of-life symptom control. BMJ Support Palliat Care.

[20] Evans R, Stone D, Elwyn G (2003). Organizing palliative care for rural populations: a systematic review of the evidence. Fam Pract.

[21] Thomas A, Menon A, Boruff J, Rodriguez AM, Ahmed S (2014). Applications of social constructivist learning theories in knowledge translation for healthcare professionals: a scoping review. Implement Sci.

[22] VanNieuwenborg L, Goossens M, De Lepeleire J, Schoenmakers B (2016). Continuing medical education for general practitioners: a practice format. Postgrad Med J.

[23] Popay J, Roberts H, Sowden A, Petticrew M, Arai L, Rodgers M, Britten N, Roen K, Duffy S (2006). Guidance on the conduct of narrative synthesis in systematic reviews. A product from the ESRC methods programme Version.

[24] Selman LE, Brighton LJ, Robinson V (2017). Primary care physicians' educational needs and learning preferences in end of life care: A focus group study in the UK. BMC Palliat Care.

[25] Pype P, Peersman W, Wens J (2014). What, how and from whom do health care professionals learn during collaboration in palliative home care: a cross-sectional study in primary palliative care. BMC Health Serv Res.

[26] Pype P, Symons L, Wens J (2014). Health care professionals' perceptions towards lifelong learning in palliative care for general practitioners: a focus group study. BMC Fam Pract.

[27] Bramer WM, de Jonge GB, Rethlefsen ML, Mast F, Kleijnen J (2018). A systematic approach to searching: an efficient and complete method to develop literature searches. J Med Libr Assoc.

[28] Bradshaw PJ (1993). Characteristics of clients referred to home, hospice and hospital palliative care services in Western Australia. Palliat Med.

[29] Hawker S, Payne S, Kerr C (2002). Appraising the evidence: reviewing disparate data systematically. Qual Health Res.

[30] Bianchi M, Bressan V, Cadorin L (2016). Patient safety competencies in undergraduate nursing students: a rapid evidence assessment. J Adv Nurs.

[31] Salins N, Ghoshal A, Hughes S, Preston N (2020). How views of oncologists and haematologists impacts palliative care referral: a systematic review. BMC Palliat Care.

[32] Waring G, Kirk S, Fallon D (2020). The impact of chronic non-specific cough on children and their families: A narrative literature review. J Child Health Care.

[33] Flemming K (2010). The use of morphine to treat cancer-related pain: a synthesis of quantitative and qualitative research. J Pain Symptom Manag.

[34] Haines CS, Thomas Z (1993). Assessing needs for palliative care education of primary care physicians: results of a mail survey. J Palliat Care.

[35] Eastaugh AN (1996). Approaches to palliative care by primary health care teams: A survey. J Palliat Care.

[36] MacLeod RD, Nash A (1991). Teaching palliative care in general practice: a survey of educational needs and preferences. J Palliat Care.

[37] Lorenc T, Petticrew M, Whitehead M (2014). Crime, fear of crime and mental health: synthesis of theory and systematic reviews of interventions and qualitative evidence.

[38] Herrmann A, Carey ML, Zucca AC, Boyd LAP, Roberts BJ (2019). Australian GPs' perceptions of barriers and enablers to best practice palliative care: a qualitative study. BMC Palliat Care.

[39] O'Connor M, Lee-Steere R (2006). General practitioners' attitudes to palliative care: A Western Australian rural perspective. J Palliat Med.

[40] O'Connor M, Breen LJ (2014). General Practitioners' experiences of bereavement care and their educational support needs: a qualitative study. BMC Med Educ.

[41] Assing Hvidt E, Søndergaard J, Ammentorp J (2016). The existential dimension in general practice: identifying understandings and experiences of general practitioners in Denmark. Scand J Prim Health Care.

[42] Becker G, Momm F, Deibert P (2010). Planning training seminars in palliative care: a cross-sectional survey on the preferences of general practitioners and nurses in Austria. BMC Med Educ.

[43] Straatman L, Miller T (2013). Paediatric palliative care: a survey of paediatricians and family practitioners. BMJ Support Palliat Care.

[44] Slort W, Blankenstein AH, Deliens L, van der Horst HE (2011). Facilitators and barriers for GP-patient communication in palliative care: a qualitative study among GPs, patients, and end-of-life consultants. Br J Gen Pract.

[45] Taubert M, Noble SI, Nelson A (2011). What challenges good palliative care provision out-of-hours? A qualitative interview study of out-of-hours general practitioners. BMJ Support Palliat Care.

[46] Rhee JJ, Zwar N, Vagholkar S (2008). Attitudes and barriers to involvement in palliative care by Australian urban general practitioners. J Palliat Med.

[47] Rhee JJ, Teo PCK, Mitchell GK, Senior HE, Tan AJH, Clayton JM. General practitioners (GPs) and end-of-life care: a qualitative study of Australian GPs and specialist palliative care clinicians [published online ahead of print, 2018 Nov 1]. BMJ Support Palliat Care. 2018:bmjspcare-2018-001531. 10.1136/bmjspcare-2018-001531. 10.1136/bmjspcare-2018-001531 30385501

[48] Meijler WJ, Van Heest F, Otter R, Sleijfer DT (2005). Educational needs of general practitioners in palliative care: outcome of a focus group study. J Cancer Educ.

[49] Jünger S, Vedder AE, Milde S, Fischbach T, Zernikow B, Radbruch L (2010). Paediatric palliative home care by general paediatricians: a multimethod study on perceived barriers and incentives. BMC Palliat Care.

[50] Magee C, Koffman J (2016). Out-of-hours palliative care: what are the educational needs and preferences of general practitioners?. BMJ Support Palliat Care.

[51] Barclay S, Wyatt P, Shore S, Finlay I, Grande G, Todd C (2003). Caring for the dying: how well prepared are general practitioners? A questionnaire study in Wales. Palliat Med.

[52] Wakefield MA, Beilby J, Ashby MA (1993). General practitioners and palliative care. Palliat Med.

[53] Shipman C, Addington-Hall J, Barclay S (2001). Educational opportunities in palliative care: what do general practitioners want?. Palliat Med.

[54] Shipman C, Addington-Hall J, Barclay S (2002). How and why do GPs use specialist palliative care services?. Palliat Med.

[55] Samaroo B (1996). Assessing palliative care educational needs of physicians and nurses: results of a survey. Greater Victoria Hospital society palliative care committee. J Palliat Care.

[56] Lloyd-Williams M, Wilkinson C, Lloyd-Williams F (2000). General practitioners in North Wales: current experiences of palliative care. Eur J Cancer Care (Engl).

[57] Johnston G, Dermott D, Philip R (2001). Educational needs in palliative care: A survey of GPs and community nurses. Eur J Gen Pract.

[58] Braun BL, Fowles JB, Forrest CB, Kind EA, Foldes SS, Weiner JP (2003). Which enrollees bypass their gatekeepers in a point-of-service plan?. Med Care.

[59] Deckers L (2018). Motivation: biological, psychological, and environmental.

[60] Schunk DH, Meece JL, Pintrich PR (2014). Motivation in education: theory, research, and applications.

[61] Tjin A, Tsoi SL, de Boer A, Croiset G, Koster AS, Kusurkar RA (2016). Unraveling motivational profiles of health care professionals for continuing education: the example of pharmacists in the Netherlands. J Contin Educ Heal Prof.

[62] van der Burgt SME, Kusurkar RA, Wilschut JA, Tsoi TA, SLNM, Croiset G, Peerdeman SM. (2018). Motivational profiles and motivation for lifelong learning of medical specialists. J Contin Educ Heal Prof.

[63] Bélanger P (2011). Theories in adult learning and education.

[64] Jarvis P (2004). Adult education and lifelong learning: theory and practice.

[65] Mezirow J, Illeris K (2009). An overview of transformative learning. Contemporary theory of learning.

[66] Keefe JW, Kiernan OB (1979). Student learning styles: diagnosing and prescribing programs.

[67] Teunissen PW, Dornan T (2008). Lifelong learning at work. BMJ..

[68] Honey P, Mumford A (1992). The manual of learning styles.

[69] Webster R. Learning styles and design: The use of ASSIST for reflection and assessment. In: Proceedings of the 2002 Annual International Conference of the Higher Education Research and Development Society of Australasia (HERDSA). Perth: Higher Education Research and Development Society of Australasia (HERDSA), Australia; 2002. http://www.herdsa.org.au/index.php.

[70] DiBartola LM (2006). The learning style inventory challenge: teaching about teaching by learning about learning. J Allied Health.

[71] Brockett RG, Hiemstra R (2018). Self-direction in adult learning: perspectives on theory, research, and practice.

[72] Fulton J (2013). Mentorship: excellence in the mundane. Br J Healthc Assist.

[73] Burgess A, van Diggele C, Mellis C (2018). Mentorship in the health professions: a review. Clin Teach.

[74] Dolcourt JL, Zuckerman G, Warner K (2006). Learners’ decisions for attending pediatric grand rounds: a qualitative and quantitative study. BMC Med Educ.

[75] Glazebrook RM, Harrison SL (2006). Obstacles and solutions to maintenance of advanced procedural skills for rural and remote medical practitioners in Australia. Rural Remote Health.

[76] Shute VJ (2008). Focus on formative feedback. Rev Educ Res.

[77] Desjardins R, Rubenson K (2013). Participation patterns in adult education: the role of institutions and public policy frameworks in resolving coordination problems. Eur J Educ.

[78] Baharudin SNA, Murad M, Mat NHH (2013). Challenges of adult learners: A case study of full time postgraduates students. Procedia Soc Behav Sci.

[79] Kolb DA (2015). Experiential learning: experience as the source of learning and development.

[80] Adams P (2006). Exploring social constructivism: theories and practicalities. Education.

[81] Brunner J, Illeris K (2009). Culture, mind, and education. Contemporary theory of learning.

[82] Williams SL, Haskard KB, DiMatteo MR (2007). The therapeutic effects of the physician-older patient relationship: effective communication with vulnerable older patients. Clin Interv Aging.

[83] Aita V, McIlvain H, Backer E, McVea K, Crabtree B (2005). Patient-centered care and communication in primary care practice: what is involved?. Patient Educ Couns.

[84] Street RL, Krupat E, Bell RA, Kravitz RL, Haidet P (2003). Beliefs about control in the physician-patient relationship: effect on communication in medical encounters. J Gen Intern Med.

[85] Groot MM, Vernooij-Dassen MJ, Crul BJ, Grol RP (2005). General practitioners (GPs) and palliative care: perceived tasks and barriers in daily practice. Palliat Med.

[86] Crawford GB, Price SD (2003). Team working: palliative care as a model of interdisciplinary practice. Med J Aust.

[87] Lee H (2004). The role of competence-based trust and organizational identification in continuous improvement. J Manag Psychol.

[88] Rowe AD, Fitness J (2018). Understanding the role of negative emotions in adult learning and achievement: A social functional perspective. Behav Sci (Basel).

[89] Fosnot CT (2005). Constructivism: theory, perspectives, and practice.

[90] Yilmaz K (2008). Constructivism: its theoretical underpinnings, variations, and implications for classroom instruction. Educ Horiz.

[91] Bound H, Lin M (2013). Developing competence at work. Vocat Learn.

[92] Hughes RG, Hughes RG (2008). Tools and Strategies for Quality Improvement and Patient Safety. Patient Safety and Quality: An Evidence-Based Handbook for Nurses.

[93] Lave J, Wenger E (1991). Situated learning: legitimate peripheral participation.

[94] Arikpo OU, Domike G (2015). Pupils learning preferences and interest development in learning. J Educ Pract.

[95] Akpan V, Igwe U, Mpamah I, Okor C (2020). Social constructivism: implications on teaching and learning. Br J Educ.

[96] Jonassen DH (2013). Transforming learning with technology: beyond modernism and post-modernism, or whoever controls the technology creates the reality. The nature of technology.

[97] Song SH, Keller JM (2001). Effectiveness of motivationally adaptive computer-assisted instruction on the dynamic aspects of motivation. Educ Technol Res Dev.

[98] Gran SF, Brænd AM, Lindbæk M, Frich JC (2016). General practitioners' and students' experiences with feedback during a six-week clerkship in general practice: a qualitative study. Scand J Prim Health Care.

[99] Downes S (2019). Recent work in Connectivism. Eur J Open Dist E-Learn.

