# Supplementary material for: Views of general practitioners on end-of-life care learning preferences: a systematic review
Source: BMC Palliat Care. 2022 Sep 21;21:162. doi: 10.1186/s12904-022-01053-9 (PMC9490975; doi:10.1186/s12904-022-01053-9)
Supplement: Supplementary file 1 — Additional file 1. Search Terms [file 12904_2022_1053_MOESM1_ESM.pdf]

Additional file 1: Search Terms

	PubMed<br>MeSH terms	CINAHL<br>MH terms	PsycINFO<br>DE terms	EMBASE<br>Emtree terms	Free text terms
**Concept 1**<br><br>**Views**	1.Attitude of health personnel<br>2.Motivation<br>3.Emotions	1.Attitude of health personnel<br>2.Motivation<br>3.Emotion<br>4.Perception<br>5.Behaviour	1. Health personnel attitudes<br>2. Motivation<br>3. Behaviour	1.Health personnel attitude<br>2. Beliefs<br>3.Emotions<br>4. Perspective<br>5. Behaviour<br>6. Opinion<br>7. Opine<br>8. Feeling<br>9. Thought<br>10. Think<br>11. Perception<br>12. Barrier<br>13. Facilitator	1.View*<br>2.Perception<br>3.Perceive*<br>4.value*<br>5.Motivation*<br>6.Opinion*<br>7.Opine*<br>8.Perspective*<br>9.Attitude*<br>10.Belief*<br>11.Feel*<br>12.Thought*<br>13.Think*<br>14.Behaviour*<br>15. Barrier*<br>16. Facilitator*
**Concept 2**<br><br>**General Practitioner**	1.General practice<br>2.General practice physician<br>3.Family physician<br>4.General practitioner<br>5.Family practice	1.General practitioner<br>2.Family physician<br>3.Primary care physician	1.Family Medicine<br>2.Family Physician	1.General practitioner<br>2.Primary care physician<br>3.Family physician<br>4.General practice physician<br>5.Primary care physician<br>6.General practice	1."General practice*"<br>2."General practitioner*"<br>3."Family physician*"<br>4."Family practice*
**Concept 3**<br><br>**Learning preference**	1.Continuing education<br>2.Continuing medical education<br>3.Competency based education	1.Continuing medical education<br>2.Continuing education<br>3.education	1.Continuing education<br>2.Medical education<br>3.Professional development<br>4.Distance education	1.Continuing medical education<br>2.Continuing education<br>3. Continuing professional development	1.Education<br>2.Learning<br>3. Continuing medical education<br>4.Continuing education<br>5.Learning style*33<br>6.Learning preference*
**Concept 4**<br><br>**End-of-life care**	1.Palliative care<br>2.Palliative medicine<br>3.Hospice<br>4.Hospice care<br>5.Hospice program<br>6.Terminal care<br>7.End of life care	1.End of life care<br>2.Terminally ill patient<br>3.palliative care	Palliative care	1.Terminal care<br>2.Palliative therapy<br>3.Hospice care<br>4.End of life care<br>5.Terminal care<br>6.Palliative care<br>7.Palliative Medicine	1.Palliat*<br>2.Hospice*<br>3.”End of life care"<br>4."supportive care"

				8.Hospice care<br>9.Hospice	
