# Supplementary material for: Views of general practitioners on end-of-life care learning preferences: a systematic review
Source: BMC Palliat Care. 2022 Sep 21;21:162. doi: 10.1186/s12904-022-01053-9 (PMC9490975; doi:10.1186/s12904-022-01053-9)
Supplement: Supplementary file 2 — Additional file 2. List of hand searched journals [file 12904_2022_1053_MOESM2_ESM.pdf]

Additional file 2. List of hand searched journals

1. BMC Palliative Care
2. BMC Family Practice
3. Journal of Palliative Medicine
4. Scandinavian Journal of Primary Health Care
5. BMC Medical Education
6. European Journal of General Practice
7. European Journal of Cancer Care
8. Journal of Cancer Education
9. BMC Health Service Research
10. BMJ Supportive and Palliative Care
11. Journal of Palliative Care
12. British Journal of General Practice
