# Supplementary material for: Views of general practitioners on end-of-life care learning preferences: a systematic review
Source: BMC Palliat Care. 2022 Sep 21;21:162. doi: 10.1186/s12904-022-01053-9 (PMC9490975; doi:10.1186/s12904-022-01053-9)
Supplement: Supplementary file 3 — Additional file 3. Quality Assessment of the studies using Hawker’s tool [file 12904_2022_1053_MOESM3_ESM.pdf]

## Additional file 3: Quality Assessment of the studies using Hawker's tool

| Author | Abstract and title | Introduction and aims | Method and data | Sampling | Data analysis | Ethics and bias | Results | Transferability/ Generalizability | Implications and usefulness | Total score |
|---|---|---|---|---|---|---|---|---|---|---|
| **Hvdit et al. (2016)** | 4 | 4 | 3 | 2 | 4 | 2 | 3 | 2 | 3 | **27** |
| **Barcley et al. (2003)** | 3 | 3 | 3 | 3 | 2 | 1 | 4 | 2 | 1 | **22** |
| **Becker et al. (2010)** | 3 | 4 | 3 | 3 | 3 | 1 | 4 | 3 | 2 | **26** |
| **Wakefield et al. (1993)** | 2 | 3 | 2 | 2 | 2 | 1 | 3 | 3 | 2 | **20** |
| **Taubert et al. (2011)** | 4 | 4 | 4 | 2 | 2 | 1 | 4 | 3 | 1 | **25** |
| **Straatman and Miller (2013)** | 4 | 4 | 3 | 2 | 2 | 1 | 3 | 3 | 2 | **24** |
| **Slort et al. (2011)** | 4 | 4 | 3 | 2 | 3 | 1 | 2 | 2 | 4 | **25** |
| **Shipman et al. (2002)** | 3 | 2 | 2 | 3 | 3 | 1 | 3 | 2 | 1 | **20** |
| **Shipman et al. (2001)** | 3 | 3 | 3 | 2 | 4 | 1 | 3 | 2 | 2 | **23** |
| **Selman et al. (2017)** | 4 | 4 | 4 | 4 | 4 | 2 | 4 | 4 | 2 | **32** |

| | | | | | | | | | |
|---|---|---|---|---|---|---|---|---|---|
| **Samaroo (1993)** | 2 | 2 | 3 | 2 | 2 | 1 | 2 | 3 | 2 | **19** |
| **Rhee et al. (2008)** | 4 | 4 | 3 | 3 | 4 | 2 | 2 | 2 | 4 | **28** |
| **Rhee et al. (2018)** | 4 | 4 | 2 | 3 | 3 | 1 | 2 | 2 | 4 | **25** |
| **Pype at al. (2014)** | 4 | 3 | 4 | 3 | 4 | 1 | 4 | 4 | 3 | **30** |
| **Pype at al. (2014)** | 4 | 4 | 4 | 4 | 4 | 2 | 3 | 3 | 3 | **31** |
| **O’Connor and Breen (2014)** | 4 | 4 | 3 | 3 | 4 | 4 | 3 | 3 | 2 | **30** |
| **O’Connor and Le-Steere (2006)** | 4 | 4 | 4 | 4 | 4 | 2 | 2 | 3 | 3 | **30** |
| **Meijler et al. (2005)** | 4 | 3 | 3 | 2 | 2 | 1 | 3 | 3 | 4 | **25** |
| **Lloyd-Williams et al. (2006)** | 2 | 2 | 2 | 3 | 2 | 3 | 1 | 3 | 2 | **20** |
| **Jhonston et al. (2001)** | 3 | 3 | 3 | 2 | 1 | 1 | 3 | 4 | 2 | **23** |
| **Hermann et al. (2019)** | 4 | 4 | 4 | 2 | 4 | 4 | 3 | 3 | 2 | **30** |
| **Junger et al. (2010)** | 4 | 3 | 4 | 3 | 3 | 2 | 3 | 3 | 2 | **27** |

| | | | | | | | | | | |
|---|---|---|---|---|---|---|---|---|---|---|
| **Maggie and Koffman (2015)** | 4 | 4 | 3 | 2 | 4 | 2 | 3 | 3 | 3 | **28** |
