# Supplementary material for: Views of general practitioners on end-of-life care learning preferences: a systematic review
Source: BMC Palliat Care. 2022 Sep 21;21:162. doi: 10.1186/s12904-022-01053-9 (PMC9490975; doi:10.1186/s12904-022-01053-9)
Supplement: Supplementary file 4 — Additional file 4. Systematic review themes and subthemes [file 12904_2022_1053_MOESM4_ESM.pdf]

Additional file 4: Systematic review themes and subthemes

Themes	Subthemes	Explanatory Narrative
**Theme 1: Motivating factors that trigger learning in end-of-life care**	Disposition to provide palliative care	Positive attitude towards helping palliative patients(**Slort et al, 2011 [44]))Rhee et al, 2018 [47]) (Jhonston et al, 2001 [57]**, **Rhee et al., 2018 [47]**), Comfort in providing palliative care (**Rhee et al., 2018 [47]**), Being humane to patient's concern(**O'Connor et al, 2014 [40]),** improve quality of life of palliative patients **(O'Connor et al, 2006 [39]), m**ore experience and higher clientele (**Barcley et al, 2003 [51]) (Rhee et al, 2008 [46]) (Junger et al, 2010 [57]), o**lder GPs are able to focus on their interest areas have more older and sicker patients(**Barcley et al, 2003 [51]**), GPs with experience in home care would prefer learning palliative care(**Barcley et al, 2003 [51]) (Jhonston et al, 2001 [57]) (Slort et al, 2011 [44])**
	Self-actualisation	Palliative care as valuable part of their care(**Meijler er al, 2005 [48])(Lloyd-William et al, 2006 [56]) (Hermann et al, 2019 [38]) (Junger et al, 2010 [49]), i**ntuition that they will be able to help the dying patient(**Junger et al, 2010 [49]**),<br>Need for an attitudinal shift from "cure to care" in order to look beyond the usual framework of a diagnosis, a therapy and making somebody better (**Pype et al, 2014 (a) [25]**), training in palliative care helps become a good physician(**Meijler et al, 2005 [48]**), Recourse to guilt from previous failures to alleviate symptoms**(O'Connor et al, 2006 [39]),** Feeling and understanding that personal loss helps empathize with patients better(O'**Connor et al, 2014 [40]**), need for coping with powerlessness, helplessness and emotional burden of caring**(Selman et al, 2017 [24] (Hermann et al, 2019 [38]) (Meijler et al, 2005[48])**
	Relevance to clinical practice	GPs trained with specialists if they felt the specialists had the skills and preference relevant to their practice(**Shipman et al, 2002 [54]**)<br>(Ability to deal with)Complexity of end of life care(**Selman et al, 2017 [24]**)<br>Deficiency in knowledge and skills(**Barcley et al, 2003 [51])**<br>Fear of treading unknown territory(**Taubert et al, 2011 [45])**<br>Confidence in prescribing analgesia triggers more learning**(Shipman et al, 2001 [53])**<br>Attrition of skills over time**(Selman et al, 2017 [24])(Pype et al, 2014 (b) [26])** due to lack of ongoing exposure to palliative care patients or due to referral to specialists or GP colleague with interest(**Selman et al, 2017 [24]**)<br>Lack of expertise in addressing specific aspects such as spiritual and religious concerns (**Assing Hvidt et al. 2016 [41]**)
	Feeling responsible	Generalist must be specialists in EOLC(**Selman et al, 2017 [24])**<br>Central to coordinating palliative care for patients in the community **(Lloyd-William et al, 2006 [56])**<br>Feel committed to caring for their patients as a trusted key person who accompanies the family for many years **(Junger et al, 2010 [49])**<br>Feeling palliative care as their core responsibility (**Pype et al, 2014 (a) [25]) (O'Connor et al, 2006 [39]**)

		Responsible for keeping patients free of pain and discomfort(**Pype et al, 2014 (b) [26])**<br>Sense of responsibility to support bereaved family (O'**Connor et al, 2014 [38])**
	Therapeutic bond	Providing care for a prolonged period for patients (**Barcley et al, 2003 [51]) (Shipman et al, 2001 [53]) (Rhee et al, 2018 [47])** from birth to end-of-life (**Junger et al, 2010 [49]**) or from diagnosis to end-of-life(O'**Connor et al, 2006 [39])** and for patients known to them in the past(**Rhee et al, 2018 [47]**) **(O'Connor et al, 2006 [39])** eases palliative care provision**(Rhee et al, 2018 [47])**<br>Strong bond that developed with long standing patient-GP relationship**(Slort et al, 2011 [44]) (Shipman et al, 2001 [53]) (O'Connor et al, 2014 [40]) (Rhee et al, 2008 [46]) (Rhee et al, 2018 [47]) (Pype et al, 2014 (a)[25]) (Lloyd-William et al, 2006 [56]) (Hermann et al, 2019 [38]**) leads to better awareness of palliative care needs of the patients**(Shipman et al, 2001 [53])**<br>Awareness of social and family background helps address patient's needs better(O'**Connor et al, 2006 [39], Connor et al, 2014[40])**<br>They believed in helping a patient as a whole person and not as a disease entity (**Junger et al., 2010 [49])** and felt the need to address the complex sufferings of patients while honoring their dignity, expectations, and wishes **(Slort et al., 2011 [44]) (Assing Hvidt et al., 2016 [41]).**

**Themes**	**Subthemes**	**Explanatory Narratives**
**Theme 2:**<br><br>**End-of-life care learning needs**	**Accessing palliative care**	Needs for identification and referral for palliative care(**Selman et al., 2017 [24]**)<br>Need for awareness about local specialist services and resources(**Selman et al., 2017 [24], Hermann et al., 2019 [38]) (Junger et al., 2010 [49]**), local systems and frameworks for navigation(**Selman et al., 2017 [24]**)<br>Clarity in roles, responsibilities and teamwork (**Selman et al., 2017 [24]**)<br>Guidelines on out-of-hours care(**Selman et al., 2017[24]**)<br>Guidelines for effective planning and provision of EOLC(**Rhee et al., 2018 [47]**)<br>Guidelines on when to refer to specialists or other resources(O'**Connor et al., 2014 [40]**)<br>Importance of and how to build a multidisciplinary team(O'**Connor et al., 2006 [39]**)

	**Pain and symptom management**	Need for training in other symptoms management(**Barcley et al., 2003 [51]) (Becker et al., 2010 [42]) (Straatman et al., 2013 [41]) (Shipman et al., 2002 [52]**, (**Pype et al., 2014(b) [26]) (O'Connor et al., 2006 [39]) (Lloyd-Williams et al., 2006[56])**<br>Palliative care emergencies**(Magee & Koffman, 2016 [50])**<br>Preference for treating symptoms that they could treat with drugs**(Jhonston et al., 2001 [57])**<br>Symptom control in non-malignant conditions(**Shipman et al., 2001 [53]) (Magee and Koffman, 2016 [50])**<br>Management of nausea/vomiting(**Shipman et al., 2001 [53]) (Meijler et al., 2005 [48]**)<br>Management of agitated delirium and complications, dyspnoea(**Samaroo et al., 1993 [55]) (Rhee et al., 2008 [46]), Magee and Koffman, 2016 [50],** (**Meijler et al., 2005 [48]**)<br>Management of hypoxia and insomnia(**Wakefield et al., 1993 [52], Meijler et al., 2005 [48]**)<br>Psychosocial issues of terminally ill patients(**Wakefield et al., 1993 [52]) (Rhee et al., 2008 [46]) (Pype et al., 2014(b) [26]**) **(O'Connor et al., 2006 [39]) (Lloyd-Williams et al., 2006 [56]**) and withdrawal symptoms(**Samaroo et al, 1993 [55]**)<br>Distinguishing adjustment disorder from sadness (**Meijler et al., 2005 [48]**)<br>Challenges in distinguishing delirium, fear of dying or anxiety(**Meijler et al., 2005[48], Jhonston et al., 2001 [57]**)<br>Handling the emotional distress of patients such as fear(**Slort et al., 2011 [44]) (O'Connor et al., 2006 [39]**) anger, demanding behavior (**Samaroo et al., 1993 [55]**)<br>Need for assessment and provision of nutrition support(**Straatman et al., 2013 [43], Meijler et al., 2005 [48]**), parenteral/PEG feeding(**Meijler et al., 2005 [48]**)<br>Other symptoms such as anorexia, fatigue, incontinence, unpleasant smell (**Jhonston et al., 2001 [57]**)<br>Need for training in addressing spiritual needs of patients(**Straatman et al., 2013 [43], Pype et al., 2014(b) [26], Jhonston et al., 2001 [57]**) and religion(**Pype et al., 2014(b)[26]**)
	**Communication skills and compassionate care**	How to facilitate decision making in life limiting conditions(**Junger et al., 2010 [49]**)<br>Training in breaking bad news to patients/family(**Shipman et al., 2001 [53]) (Pype et al., 2014(a) [25]) (O'Connor et al., 2006 [39]) (Meijler et al., 2005 [48])**<br>Communication skills in counselling patients transitioning from curative to palliative care(**Meijler et al., 2005 [48]) (Magee & Koffman, 2016 [50]**)<br>Skill building in handling difficult conversation especially in time constraints(**Selman et al., 2017 [24]**)

		Challenges of handling troublesome relationship with patients(**Slort et al., 2011 [44]**)<br>Challenges of handling patient-relative conflicts(S**lort et al., 2011 [44]**) **(O'Connor et al., 2006 [39]) (Meijler et al., 2005 [48]**)<br>Training to deal with resistant patient/family (**Selman et al., 2017 [24]**) in denial/ bargaining**(O'Connor et al., 2006 [39]) (Meijler et al., 2005 [48]) (Hermann et al., 2019 [38]**)<br>To learn how to use right attitude, diplomacy, skills/tactics to deal with difficult situation with family(O'**Connor et al., 2006 [39]), Meijler et al., 2005 [48]**)<br>Need to understand how to tailor information conveyed to family(O'**Connor et al., 2006 [39]**)<br>To tackle vulnerability and apprehensions related to communication around death and dying (**Junger et al., 2010 [49]**)
	**Address caregiver needs**	Handling the emotional distress of the relatives(**Wakefield et al., 1993 [52]) (Meijler et al., 2005 [48]**)<br>Bereavement support of caregivers(**Wakefield et al., 1993 [52] (Lloyd-William et al., 2006 [54]) (Jhonston et al., 2001 [57]) (Junger et al., 2010 [49]**)<br>Need of differentiation between grief reaction and depression(O'**Connor et al., 2014 [40]**)<br>Resolving family's anticipatory grief when in denial(O'**Connor et al., 2006 [39])**
	**Ethical and Medico-legal aspects**	To provide care in patient's best interest(**Selman et al., 2017 [24]**)<br>respecting patient's wishes and expectations(**Slort et al., 2011 [44]**)<br>To understand the ethical norms and values(**Meijler et al., 2005 [48]**)<br>How to discuss ethical aspects of care to patients/family and colleagues(**Meijler et al., 2005 [48]**)<br>Need for training in medicolegal aspects of palliative care(**Rhee et al., 2018 [47]**)
	**Teamwork**	Sharing of responsibility (**Straatman et al., 2013 [43]**)<br>Debriefing with colleagues(**Straatman et al., 2013 [43]) (Junger et al., 2010 [49]**) in an emotionally draining situation in practice(**Hermann et al., 2019 [38])**<br>To develop coping strategies(**Becker et al., 2010 [42]) (Junger et al., 2010 [49]**)<br>To deal with coping in special situation such as loss of children cared for(O'**Connor et al., 2006 [39]**) or with children of similar age as theirs (O'**Connor et al., 2006 [39]**)<br>For younger GPs who may be still developing their personal views on death and dying(O'**Connor et al., 2006 [39]**)<br>Need for mutual support from multidisciplinary team/specialists(**Junger et al., 2010 [49]**)

**Theme**	**Subtheme**	**Explanatory Narratives**

**Theme 3: Preference for a learning style**	Learning from experience and Experiential learning	Expertise grows with years of experience(**Assing Hvidt et al., 2016 [41]) (Selman et al, 2017 [22) (Pype et al., 2014(a) [25]**) **(O'Connor et al., 2006 [39]) (Lloyd-Williams et al., 2006 [56]**)<br>Expertise grows through patient-GP relationship(**Assing Hvidt et al., 2016 [41]) (Pype et al., 2014(a) [25]**)<br>Real time exposure in the workplace to palliative care helps gain confidence(**Rhee et al., 2018 [47]) (Pype et al., 2014(b) [26]**) **(O'Connor et al, 2014 [40]), Hermann et al., 2019 [38]**)<br>Intuition and gut feelings helps address certain areas such as spirituality(**Assing Hvidt et al., 2016 [41]**)<br>Need for learning through experience in a mentored environment(**Selman et al., 2017 [24]**) and from experienced general practitioners (**Selman et al, 2017 [24]) (Rhee et al, 2018 [47]) (Meijler et al, 2005 [48]) (Magee and Koffman, 2016 [50])**<br>Providing shared care with specialist palliative care team(**Selman et al., 2017 [24]) (Rhee et al., 2018 [47]**) helps shifts to more proactive style of treating (**Pype et al., 2014(a) [25]**)<br>Attending planning meetings with specialists(**Pype et al, 2014 (a)[25]**)<br>Palliative care needs to be integrated into primary care(**Rhee et al., 2018 [47]) (Hermann et al., 2019 [38])**<br>Learning from peer from different disciplines (**Becker et al., 2010 [42]) (Selman et al., 2017 [24]) (Lloyd-Williams et al., 2006 [56]) (Magee and Koffman, 2016 [50])**<br>Learning from relatives who have gone through caring for palliative patients(**Selman et al.,2017 [24]) (Pype et al., 2014(a)[25]**)
	Pragmatic learning style	Reflection on one's practice under the guidance of specialists(**Pype et al. 2014(a) [25]) (Pype et al. 2014(b) [26])**<br>Applying the principles of palliative care in practice and getting feed-back(O'**Connor et al., 2014 [40]**)<br>Learn from one's mistakes and non-success(**Taubert et al., 2011 [45]) (Pype et al, 2014(b) [26]**)<br>Reflection on one's practice of communication with specialists (**Pype et al., 2014 (b) [26])**<br>Hands-on interactional training under a mentor **(Selman et al, 2017 [24])** (**Hermann et al., 2019 [38]) (Rhee et al , 2018, [47])**<br>Problem-based learning through follow up (**Taubert et al., 2011 [45]) (Meijler et al., 2005 [48]**)<br>Case-based learning(**Junger et al.,2010 [49]) (Pype et al., 2014 (a) [25])(Magee and Koffman, 2016 [50]**) through telephonic or face-face contact(**Shipman et al., 2002 [54]), Selman et al.,2017 [24]**) sometimes also calls for instant discussion considering variability in patient needs (**Pype et al., 2014(a) [25])**<br>Cased based discussion with specialists by patient's bedside(**Samaroo et al., 1993 [55]) (Rhee et al., 2018 [47]) (Jhonston et al., 2001 [57]**)<br>Learning by auditing data(**Jhonston et al., 2001 [57]**)
	Self-learning	Internet based/text book based learning **(O'Connor et al., 2006 [39]) (Hermann et al., 2019 [38]) (Straatman et al., 2013 [43]) (Taubert et al., 2011 [45]) (Rhee et al., 2008 [46]) (Magee and Koffman, 2016 [50])**

		Computer based learning**(Jhonston et al., 2001 [57])**<br>Correspondence learning(**Straatman et al., 2013 [43]**)<br>Reference to guidelines(**Shipman et al., 2002 [54]) (Samaroo et al., 1993 [55]**)<br>E-learning as cost effective and flexible(**Selman et al., 2017 [24]**)<br>Self-learning modules(**Samaroo et al., 1993 [55]) (Rhee et al., 2008 [46]) (Pype et al., 2014(b) [26]**) **(O'Connor et al., 2014 [40]) (Junger et al., 2010 [49])**<br>Learning by researching**(Jhonston et al., 2001 [57])**
	Didactic learning	Learning by listening (**Junger et al., 2010 [49]**) (**Lloyd-William et al., 2006 [56]) (Jhonston et al., 2001 [57])** and observing(for topics like team building, religious and psychological topics)(**Pype et al., 2014(a) [25]), (O'Connor et al., 2014[40]**)

**Theme**	**Subtheme**	**Explanatory Narratives**
**Theme 4:**<br>**Perceived facilitators of learning**	Amicable learning environment	Learning in a respectful environment that will be non-intimidating or non-judgemental (**Pype et al, 2014(a) [25])** Learning that happens in a safe and trusted environment(**Pype et al, 2014(b) [26]**) where mentors are not be reprimanding **(Pype et al, 2014 (a) [25]) (Pype et al, 2014 (b)[26])**<br>Learning that does not expose their deficiency to their patients(**Pype et al, 2014(a) [25])**
	Preference for mentor and peer group	Multidisciplinary mentors(**Pype et al., 2014(a) [25]) (Lloyd-William et al., 2006 [56]) (Magee and Koffman, 2016 [50])** including GP colleagues**(Meijler et al., 2005 [48]) (Magee & Koffman, 2016 [50])** palliative physicians and palliative care nurse(**Magee and Koffman, 2016 [50])**<br>Small multidisciplinary peer group(**Becker et al., 2010 [42]) (Magee and Koffman, 2016 [50]) (Pype et al., 2014(a) [25]) (Hermann et al., 2019 [38])**
	Preference for timing of training	Evening courses(**Becker et al., 2010 [42]**)<br>Weekend courses(**Becker et al., 2010 [42] (Straatman et al., 2013 [43]**)<br>Half day workshop(**Samaroo et al., 1993 [55]**)<br>Quarterly topical in-service training(**Samaroo et al., 1993 [55]**)<br>One day on-site training(**Samaroo et al., 1993 [55]**)<br>Quarterly case rounds(**Samaroo et al., 1993 [55]**)<br>Learning as being ongoing and lifelong**(Pype et al., 2014(a) [25]) (Hermann et al., 2019 [38])**<br>Out of business hours**(Hermann et al., 2019 [38])**
	Preference for feedback	Patient and family feedback was most preferred but if done sensitively (**Selman et al., 2017 [24]) (O'Connor et al., 2014 [40])**<br>Timing of the feedback was important and wanted it filled by familiar face as otherwise they feared low return rates(through posts) **(Selman et al., 2017 [24])**<br>Apprehension of negative feedback from patient and family (**Selman et al., 2017 [24])**<br>Preferred to avoid feedback from dying patient or a family as it would cause discomfort to them (**Selman et al., 2017 [24]**)<br>Preferred feedback from specialists **(Pype et al. 2014(a) [25])**<br>Feedback from members in the team in a trusted environment**(Pype et al., 2014(b) [26])**

		Behavioural assessment using videotaping or simulation**(Selman et al., 2017 [24]) (Jhonston et al., 2001 [57])** was questioned as GPs felt this could be rehearsed<br>Self-assessment forms were considered of limited use although GPs filled it sincerely **(Selman et al., 2017 [24])**<br>Self-assessment forms were filled if they were not too lengthy(**Selman et al., 2017 [24]**)

**Theme 5:**<br><br>**Perceived Barriers of Learning**	**Subtheme**	**Explanatory Narratives**
**Dispositional barriers**	**Diffidence to discuss end of life care**	Guilt or hopelessness of not having confidence in caring for dying patient(**Junger et al., 2010 [49]**)<br>Taboo about discussion around death and dying**(Assing Hvidt et al, 2016 [41]) (Junger et al, 2010 [49])**<br>Fear of causing discomfort to patient and family about aspects such as spirituality and religion(**Assing Hvidt et al, 2016 [41]**)<br>Fear of invading the private space- spiritual space is private to patient/family(**Assing Hvidt et al, 2016 [41])**<br>Fear of blame or medico-legal recrimination for wrong doing (**Taubert et al, 2011 [45]) (Meijler et al, 2005 [48]**)
**Situational barriers**	**Challenges at an individual level**	Younger GPs with less years of experience have less autonomy to focus on specific specialty (**Assing Hvidt et al.,2016 [41]) (Barcley et al, 2013 [51]) (Rhee et al, 2008 [46]**)<br>Insecurity to broach their deficiency in knowledge can inhibit their inclination to learn(**Taubert et al, 2011 [45]**)<br>Lower incidence of palliative care patients results in less motivation of GPs to learn(**Shipman et al, 2002 [54]) (Selman et al, 2017 [24]**)<br>Less sick patients had less palliative care needed**(Rhee et al, 2018 [47])**<br>Perception of having sufficient knowledge(**Shipman et al, 2002 [54]**) and no new developments in the field of palliative care(O'**Connor et al, 2006 [39]) (O'Connor et al, 2014 [40]**)<br>Personal/family commitment gives less time to training(**Rhee et al, 2008 [46]**)<br>Care as being emotionally draining(O'**Connor et al, 2006 [39]**)
	**Resource constraints**	General practitioners felt that there was a lack of systematic or standardised guidelines to help address palliative care needs (**Assing Hvidt et al, 2016 [41]) (Hermann et al, 2019 [38]**) or knowledge of resources where their patients could be referred for specialised care (**Assing Hvidt et al, 2016 [41]**). Rural general practitioners or general practitioners who had solo or small practice had less support system that limited their access to training (O'**Connor et al, 2006 [37] (Shipman et al, 2001 [53]) (Junger et al, 2010 [49]) (Barcley et al, 2003 [51])**
**Professional barriers**	**Challenges at professional level**	Excess work pressure with resultant lack of time as a constraint to learning**(Shipman et al, 2002 [53]) (Assing Hvidt et al, 2016 [41]) (Rhee et al, 2008 [46]) (Pype et al, 2014(a) [25]) (Jhonston et al, 2001 [57]) (Hermann, et al, 2019 [38]) (Selman et al, 2017 [24]) (O'Connor et al, 2006 [39]) (Meijler et al, 2005 [48])**

		Remuneration incommensurate with the work done(**Rhee et al, 2008 [46]) (Hermann et al, 2019 [47]**)<br>Having to self-fund their course(**Jhonston et al, 2001 [57]**)<br>Self-expense for organizing locum in their absenteeism from work(**Jhonston et al, 2001 [57]**)<br>Temporary job or temporary time/shift patterns at work gives less opportunity to learning on job or reflective learning (O'**Connor et al, 2006 [39]**) (**Taubert et al, 2011 [45])**
	**Disempowerment of General practitioners**	Referral to specialists or experienced GPs(**Selman et al, 2017 [24])** and when specialists take over the care (**Rhee et al, 2008 [46]) (Pype et al, 2014 (a)[25])** leads to loss of control **(O'Connor et al, 2006 [39]**)<br>Lack of trust in GPs by specialists(**Selman et al, 2017 [24]**)<br>Do not consider GPs as doing a worthwhile job in EOLC(**Selman et al, 2017 [24]**)<br>GPs do not feel recognized for their job(**Hermann et al, 2019 [38]) (Meijler et al, 2005 [48])** or appreciated for their work(**Hermann et al, 2019 [38]**)<br>Patients lack trust in general practitioner's ability to care(**Hermann et al, 2019 [38]**)<br>Inability to address patient's needs instilled a fear of strain in doctor-patient relationship(**Meijler et al, 2005 [48]**)
	**Conflict in care provision**	Lack of clarity in roles and responsibilities **(Pype et al, 2014 (a)[25]) (O'Connor et al, 2014 [40])** (**Shipman et al, 2002[54])**<br>Fear of confronting specialists in a conflict in treatment(**Shipman et al, 2002 [54]**)<br>Fear of being reprimanded/accused by specialists(**Shipman et al, 2002 [54]**)<br>Delay in response from specialists (**Shipman et al, 2002 [54])**<br>Past bitter experience with specialists can inhibit future interaction with specialists(**Shipman et al, 2002 [54])**
**Academic barrier**	**Training not aligned with clinical practice**	Most trainings being oncology focused gives them less opportunity to learn non malignant palliative care which comprises major part of their practice(**Shipman et al, 2001 [53]**)<br>Lack of locally based training resources(**Jhonston et al, 2001 [57]) (Hermann et al, 2019 [38])** or counseling support**(O'Connor et al, 2014 [40])**<br>Lack of consistency in training due to poor communication and documentation by specialists(O'**Connor et al, 2014 [40]**)<br>Most trainings are in-hour to which GPs have less exposure (**Taubert et al, 2011 [45]**)<br>Lack of same structure and resources in the community as in the hospital leads to inability to replicate the training acquired in the hospital back in the community (**Pype et al, 2014 (a) [25])**<br>Lack of accreditation of palliative care(**Hermann et al, 2019 [38]**)<br>Lack of exposure to community during training period(**Selman et al, 2017 [24]**)<br>Training also depends on trainer's inclination to palliative care(**Selman et al, 2017 [24]**)
