# Supplementary material for: Views of general practitioners on end-of-life care learning preferences: a systematic review
Source: BMC Palliat Care. 2022 Sep 21;21:162. doi: 10.1186/s12904-022-01053-9 (PMC9490975; doi:10.1186/s12904-022-01053-9)

Additional file 5: Thematic diagram of General Practitioner's End-of-Life Care learning preferences

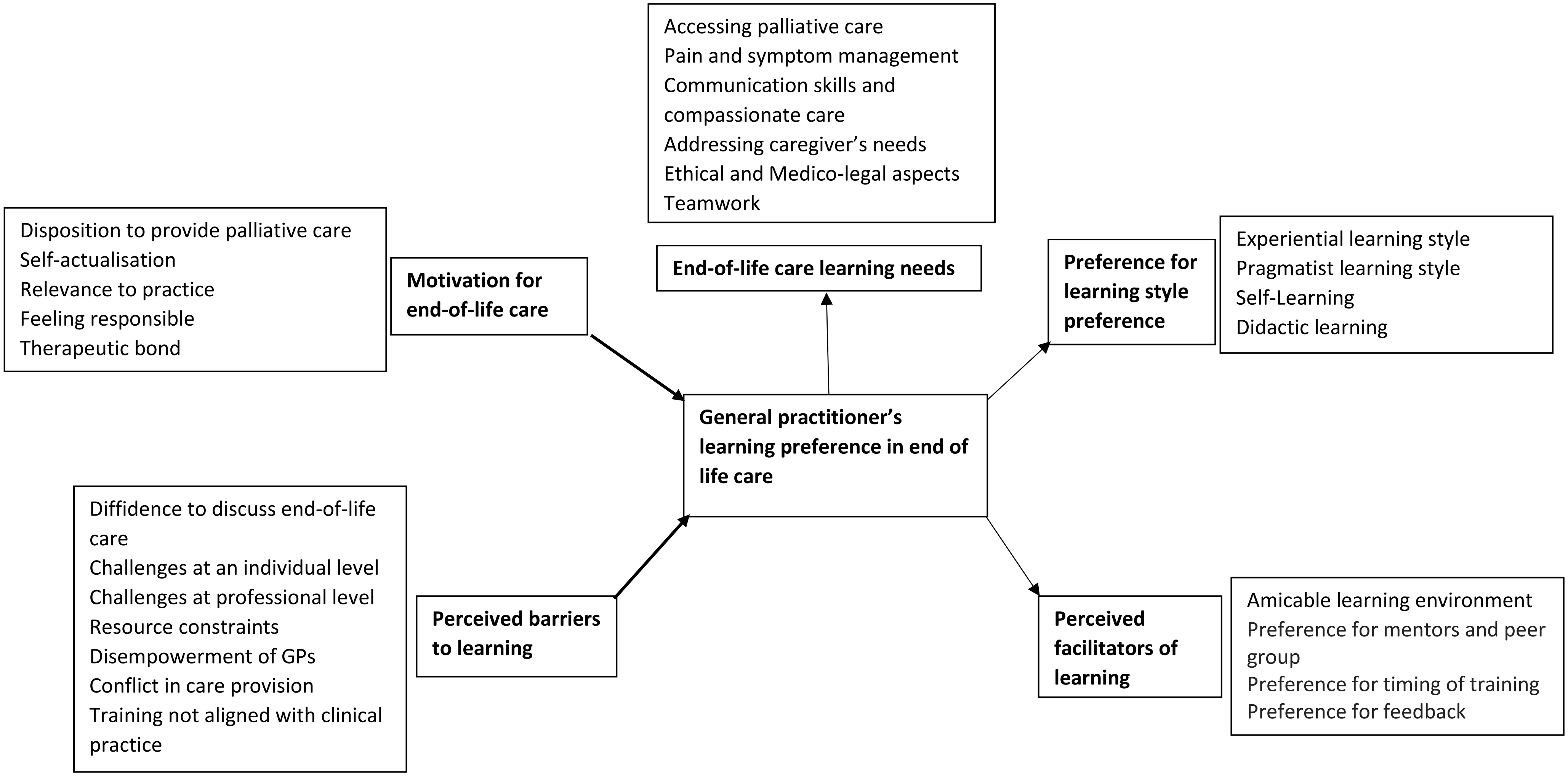

Supplement: Supplementary file 5 — Additional file 5. Thematic diagram of General Practitioner’s End-of-Life Care learning preferences [file 12904_2022_1053_MOESM5_ESM.pdf]
