# Supplementary material for: Views of general practitioners on end-of-life care learning preferences: a systematic review
Source: BMC Palliat Care. 2022 Sep 21;21:162. doi: 10.1186/s12904-022-01053-9 (PMC9490975; doi:10.1186/s12904-022-01053-9)
Supplement: Supplementary file 6 — Additional file 6. Hawker’s tool [file 12904_2022_1053_MOESM6_ESM.pdf]

Additional file 6: Hawker's tool

Assessment Form 1: Reject/Accept

Author(s):
Date of Publication:
Abbreviated Title:
Reviewer:

Relevance to Research Questions

APPENDIX C

Author and title: ______________________
Date: ______________________

	Good	Fair	Poor	Very Poor	Comment
1. Abstract and title					
2. Introduction and aims					
3. Method and data					
4. Sampling					
5. Data analysis					
6. Ethics and bias					
7. Findings/results					
8. Transferability/generalizability					
9. Implications and usefulness					
Total					

Appendix 6: Hawker's tool

APPENDIX D

1. Abstract and title: Did they provide a clear description of the study?

Good	Structured abstract with full information and clear title.
Fair	Abstract with most of the information.
Poor	Inadequate abstract.
Very Poor	No abstract.

2. Introduction and aims: Was there a good background and clear statement of the aims of the research?

Good	Full but concise background to discussion/study containing up-to-date literature review and highlighting gaps in knowledge.<br>Clear statement of aim AND objectives including research questions.
Fair	Some background and literature review.<br>Research questions outlined.
Poor	Some background but no aim/objectives/questions, OR<br>Aims/objectives but inadequate background.
Very Poor	No mention of aims/objectives.<br>No background or literature review.

3. Method and data: Is the method appropriate and clearly explained?

Good	Method is appropriate and described clearly (e.g., questionnaires included).<br>Clear details of the data collection and recording.
Fair	Method appropriate, description could be better.<br>Data described.
Poor	Questionable whether method is appropriate.<br>Method described inadequately.<br>Little description of data.
Very Poor	No mention of method, AND/OR<br>Method inappropriate, AND/OR<br>No details of data.

4. Sampling: Was the sampling strategy appropriate to address the aims?

Good	Details (age/gender/race/context) of who was studied and how they were recruited.<br>Why this group was targeted.<br>The sample size was justified for the study.<br>Response rates shown and explained.
Fair	Sample size justified.<br>Most information given, but some missing.
Poor	Sampling mentioned but few descriptive details.
Very Poor	No details of sample.

5. Data analysis: Was the description of the data analysis sufficiently rigorous?

Good	Clear description of how analysis was done.<br>Qualitative studies: Description of how themes derived/ respondent validation or triangulation.<br>Quantitative studies: Reasons for tests selected hypothesis driven/ numbers add up/statistical significance discussed.
Fair	Qualitative: Descriptive discussion of analysis.<br>Quantitative.
Poor	Minimal details about analysis.
Very Poor	No discussion of analysis.

Appendix 6: Hawker's tool

6. Ethics and bias: Have ethical issues been addressed, and what has necessary ethical approval gained? Has the relationship between researchers and participants been adequately considered?

Good	Ethics: Where necessary issues of confidentiality, sensitivity, and consent were addressed.
	Bias: Researcher was reflexive and/or aware of own bias.
Fair	Lip service was paid to above (i.e., these issues were acknowledged).
Poor	Brief mention of issues.
Very Poor	No mention of issues.

7. Results: Is there a clear statement of the findings?

Good	Findings explicit, easy to understand, and in logical progression.
	Tables, if present, are explained in text.
	Results relate directly to aims.
	Sufficient data are presented to support findings.
Fair	Findings mentioned but more explanation could be given.
	Data presented relate directly to results.
Poor	Findings presented haphazardly, not explained, and do not progress logically from results.
Very Poor	Findings not mentioned or do not relate to aims.

8. Transferability or generalizability: Are the findings of this study transferable (generalizable) to a wider population?

Good	Context and setting of the study is described sufficiently to allow comparison with other contexts and settings, plus high score in Question 4 (sampling).
Fair	Some context and setting described, but more needed to replicate or compare the study with others, PLUS fair score or higher in Question 4.
Poor	Minimal description of context/setting.
Very Poor	No description of context/setting.

9. Implications and usefulness: How important are these findings to policy and practice?

Good	Contributes something new and/or different in terms of understanding/insight or perspective.
	Suggests ideas for further research.
	Suggests implications for policy and/or practice.
Fair	Two of the above (state what is missing in comments).
Poor	Only one of the above.
Very Poor	None of the above.
